# No evidence for genotype-treatment interactions with breast cancer endocrine therapy adverse effects in UK Biobank

**DOI:** 10.1038/s41523-026-00923-2

**Published:** 2026-02-26

**Authors:** Kinan Mokbel, Michael N. Weedon, Victoria Moye, Katherine S. Ruth, Leigh Jackson

**Affiliations:** 1 Health and Care Professions Department, Faculty of Health and Life Sciences, University of Exeter Medical School, Exeter, UK; 2 Clinical and Biomedical Sciences Department, Faculty of Health and Life Sciences, University of Exeter Medical School, Exeter, UK

**Keywords:** Cancer, Genetics, Diseases, Oncology, Risk factors

## Abstract

Breast cancer is the most commonly diagnosed cancer worldwide. Earlier studies have demonstrated that breast cancer patients with particular genomic variants are more susceptible to adverse drug effects (ADEs) when they are receiving endocrine therapy. However, to establish a robust body of evidence with regard to the potential utility and predictive value of these variants, findings from these reports require replication. This study aimed to validate previously reported associations between genomic variants and medically important adverse drug effects (MIADEs) using UK Biobank (UKBB). In 2729 female participants who had received endocrine therapy in the UKBB, no statistically significant genotype-treatment interactions were observed for the outcomes examined after correction for multiple testing. Power was limited for modest interactions involving low-frequency variants and less frequent outcomes, whereas power was high to detect larger interaction effects in common-variant scenarios. Accordingly, the findings do not provide robust evidence to support previously reported pharmacogenomic associations in this dataset, and current evidence does not support the use of pharmacogenomic testing for individualised endocrine therapy selection in clinical practice.

## Introduction

Breast cancer is the most common malignancy worldwide, with approximately 2.3 million new cases diagnosed each year^1^. Seventy to eighty per cent of breast cancer cases are hormone receptor-positive (HR+) cases, for which endocrine therapy, including tamoxifen and aromatase inhibitors (AIs), plays a pivotal role in preventing recurrence and improving survival. For HR+ early breast cancer, AIs have shown greater efficacy than Tamoxifen as adjuvant therapy in postmenopausal women, whereas tamoxifen with or without ovarian function suppression is still the appropriate endocrine therapy in premenopausal women^2^. Nevertheless, both tamoxifen and AIs significantly reduce relapse rates, increase survival rates and reduce breast cancer mortality when they are administered for a 5–10-year period^3^. Breast cancer remains, however, the leading cause of cancer-related death among women, primarily due to metastasis and recurrence^4^. Although endocrine therapy has been proven effective for many years, not all women experience its benefits because of a lack of adherence. Endocrine therapy-related adverse drug effects (ADEs), which impact 30–70% of patients, are the main predictors of poor adherence and persistence^5^. These symptoms mainly include musculoskeletal, vasomotor, metabolic, vascular, vulvovaginal and endometrial symptoms^6,7^. Hence, interventions to improve breast cancer prognosis should encompass measures to prevent the ADEs associated with endocrine therapy.

Emerging evidence suggests that patients with certain genomic variants may be susceptible to clinical toxicity outcomes when undergoing endocrine therapy^8,9^, but prior studies have produced conflicting results^10,11^. These findings have been marred by substantial heterogeneity and further limited by suboptimal methodological rigour and small sample sizes. Furthermore, the majority of these pharmacogenomic studies were mainly focused on cohorts of breast cancer patients with specific cancer stages or comorbidities. Thus, it is crucial to establish the reliability of these findings by replicating them successfully in independent and well-designed large cohorts. This study aimed to replicate the previously reported associations between genomic variants and medically important adverse drug effects (MIADEs) associated with endocrine therapy in female participants in the UK Biobank (UKBB)^12^. This replication effort is a step forward in determining whether genetic variants need to be considered in clinical practice as a means of preventing MIADEs related to endocrine therapy.

## Results

### Twenty-four studies reported associations between variants and MIADEs

We identified 41 genomic variants significantly associated with MIADEs related to endocrine treatment across 24 studies^13–36^ (Table 1). These studies were categorised by system organ class, treatment modality and number of variants examined (Table 2). Musculoskeletal and reproductive MIADEs were the most studied outcomes, examined in 42% and 21% of the studies, respectively (Fig. S1). The 41 variants were identified in 19 genes, with *CYP19A1*, *ESR1* and *CYP2D6* being the most frequently examined, accounting for 27%, 15% and 12% of the total number of analysed variants, respectively (Fig. S2). Notably, only 12.5% of the studies appropriately incorporated statistical interactions, effect modifications or interaction effects models in their analyses (Table S1).

**Table 1:** Main characteristics of the studies included in this analysis

Study first author & reference	Year	Drug(s)	Genes	SNV ID or alternate names	Adverse drug event or parameter	Menopausal status	Sample	Study size/ ethnicity or country	Study type
Al-Mamun^13^	2017	Tamoxifen	*UGT2B7* *CYP2D6*	UGT2B7*2 CYP2D6*4 CYP2D6*10	Depression	Pre-, peri- and postmenopausal	Blood	(*N* = 388), Bangladesh	Cohort
Argalacsova^14^	2017	Tamoxifen	*ABCB1*	rs1045642	Endometrial hyperplasia or cancer	Pre- and postmenopausal	Blood	(*N* = 258), Czech Republic	Cohort
Baatjes^25^	2020	Anastrozole; Exemestane; Letrozole	*CYP19A1*	rs10046	Bone loss (bone mineral density) at total hip, lumbar spine	Postmenopausal	Blood	(*N* = 72), South Africa	Nested study within a prospective cohort
Chu^30^	2007	Tamoxifen	*CYP3A4*	CYP3A4*1B	Endometrial cancer	Pre- and postmenopausal	Blood	(*N* = 126) (cases = 63; controls = 63), European	Case/control
Dieudonné^31^	2014	Tamoxifen	*CYP2D6*	rs3892097	Double endometrial thickness/Hyperplasia	Postmenopausal	Blood	(*N* = 184), Belgium	Cohort
Garber^32^	2010	Tamoxifen	*F5*	rs6025	Thromboembolic events	Pre-, peri- and postmenopausal	Blood	(*N* = 412) (cases = 141; controls = 271), United States (mixed)	Case/control
Hartmaier^33^	2012	Tamoxifen	*NCOA1*	rs1804645	Bone loss (bone mineral density) at lumbar spine	Pre-, peri- and postmenopausal	Blood	(*N* = 111), Mostly Caucasian	A substudy of prospective observational cohort
Koukouras^34^	2012	Anastrozole; Exemestane; Letrozole	*ESR1*	XbaI (rs9340799)	Endometrial thickness	Postmenopausal	Blood	(cases = 87; controls = 80)	Prospective case-control study
		Anastrozole; Exemestane; Letrozole	*ESR1*	XbaI (rs9340799) PvuII (rs2234693)	LDL serum levels, Triglycerides serum levels				
Kovac^35^	2015	Tamoxifen	*F5*	rs6025	Venous thromboembolism	Pre- and postmenopausal	Blood	(*N* = 150) (cases = 50; controls = 100), Serbia	Prospective case-control study
Leyland-Jones [2]^36^	2015	Letrozole	*ESR1* *ESR2*	rs2077647 rs4986938	Grade 3–4 osteoporosis or bone fractures	Postmenopausal	FFPE primary breast cancer tissue #	(*N* = 1940) [Predominantly European Caucasian population]	Post hoc of randomised, double-blind phase III trial
Leyland-Jones [1]^15^	2015	Tamoxifen	*CYP19A1*	rs4646	Grade 3–4 osteoporosis or bone fractures	Postmenopausal	FFPE primary breast cancer tissue #	(*N* = 4580) patients on tamoxifen and/or letrozole [Predominantly European Caucasian population]	Post hoc of randomised, double-blind trial
		Letrozole	*CYP19A1*	rs936308					
Mazzuca^16^	2016	Anastrozole; Letrozole	*CYP19A1*	rs4646	Osteoporosis (bone mineral density) at the lumbar spine and proximal femur	Postmenopausal	Blood	(*N* = 45), Italy	Retrospective cohort
Miranda^17^	2021	Tamoxifen	*CYP3A5* *CYP3A5*	CYP3A5*3	Endometrial hyperplasia	Pre- and postmenopausal	Blood	(*N* = 162), Chilean	Retrospective case-control study
Napoli^18^	2013	Anastrozole; Exemestane; Letrozole	*CYP19A1*	rs700518	Bone loss (bone mineral density) at the spine, hip and femur	Postmenopausal	Blood	(*N* = 97), United States	Longitudinal prospective observational study
Ntukidem^19^	2008	Tamoxifen	*ESR1* *ESR2*	XbaI (rs9340799) ER-β (rs4986938)	Total cholesterol, Triglycerides, HDL-cholesterol, LDL-cholesterol	Postmenopausal	Blood	(*N* = 134), 92% Caucasians	A substudy of prospective observational cohort
Oesterreich^20^	2015	Letrozole; Exemestane	*CYP19A1* *ESR1* *ESR2* *HTR2A*	rs6493497 rs4870061 rs9322335 rs10140457 rs3742278 rs2813543	Bone loss [T score at the spine or hip]	Postmenopausal	Blood	(*N* = 123 on letrozole; *N* = 101 on exemestane), United States	Post hoc of prospective randomised trial
Ohnishi^21^	2005	Tamoxifen	*CYP17*	rs743572	Hepatic steatosis	Pre- and postmenopausal	Blood	(*N* = 180), Japan	Cohort
Onitilo^22^	2009	Tamoxifen	*ESR1*	XbaI (rs9340799)	Venous thromboembolism [DVT/PE]	N/R	Blood	(*N* = 219), white females, United States	Population-based cohort study
Rodríguez-Sanz^23^	2015	Anastrozole; Exemestane; Letrozole	*CYP11A1*	rs4077581 rs11632698 rs900798	Bone loss (bone mineral density) at femoral neck	Postmenopausal	Blood	(*N* = 307), Spain	Prospective, observational, clinical cohort study
Santa-Maria^24^	2016	Letrozole	*CYP19A1*	rs1062033 rs1008805 rs10046 rs2289105 rs3759811 rs700518 rs4775936 rs749292 rs4646 rs1008805	HDL, Triglycerides	Postmenopausal	Blood	(*N* = 303), United States	Sub-analysis of a prospective multicenter randomised observational open-label trial
Wang^26^	2013	Anastrozole; Letrozole	*ESR1*	rs2234693 rs9340799	Grade ≥2 MS-ADEs	Postmenopausal	Blood	(*N* = 436) (cases = 206; controls = 230), East Asian	Case/control
Wang^27^	2015	Anastrozole; Letrozole	*OPG* *RANKL*	rs2073618 rs7984870	Lumbar spine T-score or bone loss (bone mineral density) at lumbar spine	Postmenopausal	Blood	(cases = 208; controls = 212), East Asian	Case/control
		Anastrozole; Letrozole	*OPG* *RANKL*	rs2073618 rs7984870	Grade ≥3 MS-ADEs	Postmenopausal	Blood	(cases = 208; controls = 212), East Asian	Case/control
Weng^28^	2013	Tamoxifen	*E2F7* *PTCSC2* *POLQ SLC22A23*	rs310786 rs10983920 rs9862879 rs4959825	Bone loss (bone mineral density) at the spine and hip	Pre-, peri- and postmenopausal	Blood	(*N* = 245) European/Caucasian	Post hoc of open-label, prospective observational trial
Wickramage^29^	2017	Tamoxifen	*CYP2D6*	CYP2D6*41	Fatty liver	Pre- and postmenopausal	Blood	(*N* = 24), Sri Lanka	Retrospective cohort

*FFPE* Formalin-fixed, paraffin-embedded.

**Table 2:** Studies included in this analysis grouped by system organ class, treatment modality, number of SNVs and related MIADEs

System Organ Class	Endocrine agent & variants (*n*)	Adverse effects	Studies (*n*)	References
Musculoskeletal disorders	Tamoxifen (*n* = 7), Aromatase Inhibitors (*n* = 18)	BMD^a^, *T*-score^a^, Bone fractures, Osteoporosis^a^, MS-ADEs	(*n* = 10)	^15,16,18,20,23,25–28,33^
Metabolism disorders	Tamoxifen (*n* = 2), Aromatase Inhibitors (*n* = 11)	Hypercholesterolaemia^a^, Hypertriglyceridaemia^a^	(*n* = 3)	^19,24,34^
Hepatobiliary disorders	Tamoxifen (*n* = 4)	Hepatosteatosis	(*n* = 2)	^21,29^
Vascular disorders	Tamoxifen (*n* = 3)	Thromboembolic events (incl. DVT, PE)	(*n* = 3)	^22,32,35^
Reproductive system disorders	Tamoxifen (*n* = 4), Aromatase Inhibitors (*n* = 1)	Endometrial cancer, Endometrial Hyperplasia	(*n* = 5)	^14,17,30,31,34^
Psychiatric disorders	Tamoxifen (*n* = 3)	Depression	(*n* = 1)	^13^

^a^Continuous measurements or binary outcomes derived from baseline measurements.

### 2729 women in the UK Biobank were receiving endocrine therapy

**Fig. 1:**
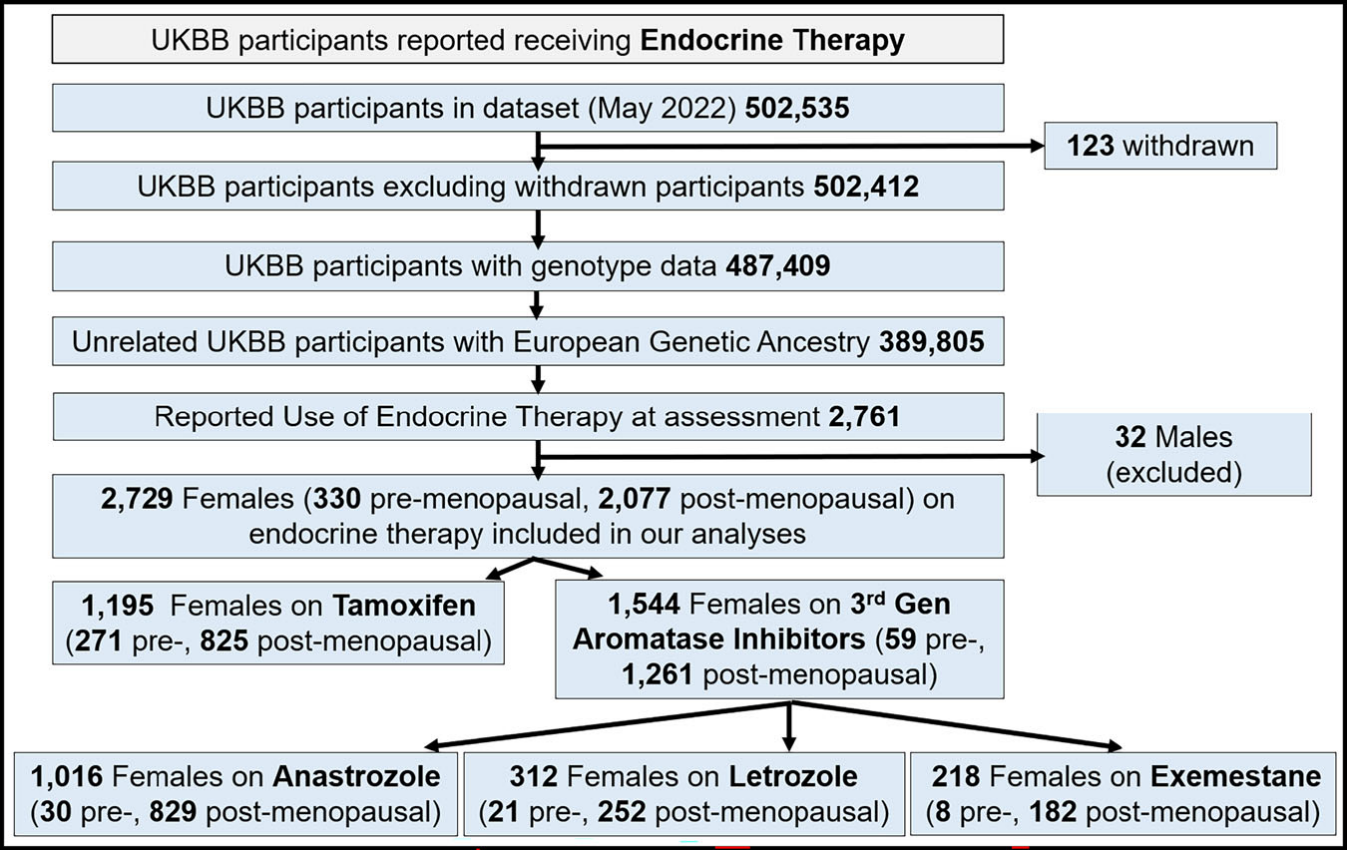
The UK Biobank cohort of patients who reported taking endocrine agents. A flow chart demonstrating the number of UKBB female participants with sufficient genomic and treatment data included in the analyses.

In the UKBB, we identified 2729 female participants who received endocrine therapy (mean age = 59.2 years). Of these, 1195 were on tamoxifen (271 premenopausal and 825 postmenopausal) and 1544 were on AIs (59 premenopausal and 1261 postmenopausal). Among the AI group, 1016 were on Anastrozole, 312 were on letrozole, and 218 were on exemestane (Fig. 1). The participant characteristics are detailed in Table 3.

**Table 3:** Characteristics of the UK Biobank female participants taking endocrine agents*

	Treatment	Endocrine therapy				The rest of the UKBB female Participants			
Main characteristics	Menopausal status	Premenopausal (*n* = 330)	Postmenopausal (*n* = 2077)	Other (*n* = 322)	Total (*n* = 2729)	Premenopausal (*n* = 55,819)	Postmenopausal (*n* = 132,736)	Other (*n* = 19,164)	Total (*n* = 207,719)
	Age (years) mean (SD)	49.6 (5.1)	60.2 (6.2)	62.7 (5.1)	59.2 (7)	47.4 (4.5)	60.2 (5.7)	62.6 (5.2)	57 (7.9)
	BMI (kg/m^2^) mean (SD)	25.7 (4.4)	27.4 (4.9)	27.7 (5.1)	27.2 (4.9)	26.4 (5.3)	27.1 (5)	27.6 (5.2)	27 (5.1)
Continuous outcomes	Bone mineral density (g/cm^2^) mean (SD)	0.51 (0.11)	0.49 (0.11)	0.48 (0.1)	0.49 (0.11)	0.55 (0.12)	0.5 (0.12)	0.5 (0.12)	0.52 (0.12)
	T-score mean (SD)	−0.58 (0.97)	−0.83 (1.02)	−0.96 (0.88)	−0.82 (1)	−0.24 (1.04)	−0.68 (1.06)	−0.73 (1.06)	−0.57 (1.07)
	Total cholesterol [mmol/L] mean (SD)	5.3(1.03)	5.81 (1.12)	5.89 (1.19)	5.76 (1.13)	5.45 (0.97)	6.07 (1.13)	6 (1.17)	5.9 (1.12)
	Triglycerides [mmol/L] mean (SD)	1.53 (0.92)	1.77 (0.97)	1.84 (1.02)	1.75 (0.98)	1.3 (0.74)	1.64 (0.87)	1.7 (0.88)	1.55 (0.85)
	LDL [mmol/L] mean (SD)	3.13 (0.79)	3.57 (0.87)	3.61 (0.92)	3.52 (0.88)	3.33 (0.76)	3.76 (0.87)	3.71 (0.91)	3.64 (0.87)
	HDL [mmol/L] mean (SD)	1.59 (0.39)	1.57 (0.37)	1.55 (0.39)	1.57 (0.38)	1.56 (0.36)	1.62 (0.38)	1.59 (0.39)	1.6 (0.38)
Binary outcomes	Osteoporosis [*n* (%)]	4 (1.31)	56 (3.03)	2 (0.7)	62 (2.54)	177 (0.36)	2452 (2.06)	442 (2.58)	3071 (1.65)
	Bone fractures [*n* (%)]	31 (9.84)	138 (7.04)	34 (11.22)	203 (7.88)	1935 (3.59)	8727 (6.92)	1577 (8.71)	12,239 (6.17)
	MS-ADEs [*n* (%)]	0(0)	2 (0.1)	1 (0.31)	3 (0.11)	21 (0.04)	115 (0.09)	17 (0.09)	153 (0.07)
	Hepatosteatosis [*n* (%)]	4 (1.21)	49 (2.37)	5 (1.56)	58 (2.13)	542 (0.97)	2113 (1.6)	390 (2.04)	3045 (1.47)
	Thromboembolic events [*n* (%)]	7 (2.16)	70 (3.54)	22 (7.33)	99 (3.8)	502 (0.91)	2871 (2.24)	528 (2.86)	3901 (1.93)
	Venous thromboembolism (DVT/PE) [*n* (%)]	6 (1.85)	48 (2.42)	12 (3.99)	66 (2.53)	311 (0.57)	1706 (1.33)	288 (1.55)	2305 (1.14)
	Endometrial cancer [*n* (%)]	4 (1.21)	27 (1.3)	4 (1.25)	35 (1.29)	183 (0.33)	784 (0.59)	109 (0.57)	1076 (0.52)
	Endometrial hyperplasia [*n* (%)]	10 (3.05)	8 (0.39)	1 (0.31)	19 (0.7)	203 (0.36)	241 (0.18)	45 (0.24)	489 (0.24)
	Depression [*n* (%)]	14 (4.62)	84 (4.41)	19 (6.83)	117 (4.7)	1934 (3.78)	4808 (3.91)	1003 (5.77)	7745 (4.04)

^*^Values are presented as the mean (SD) [range min-max] or [number of cases (%)].

### No previously reported genotype‒treatment interactions replicated in the UK Biobank

**Fig. 2:**
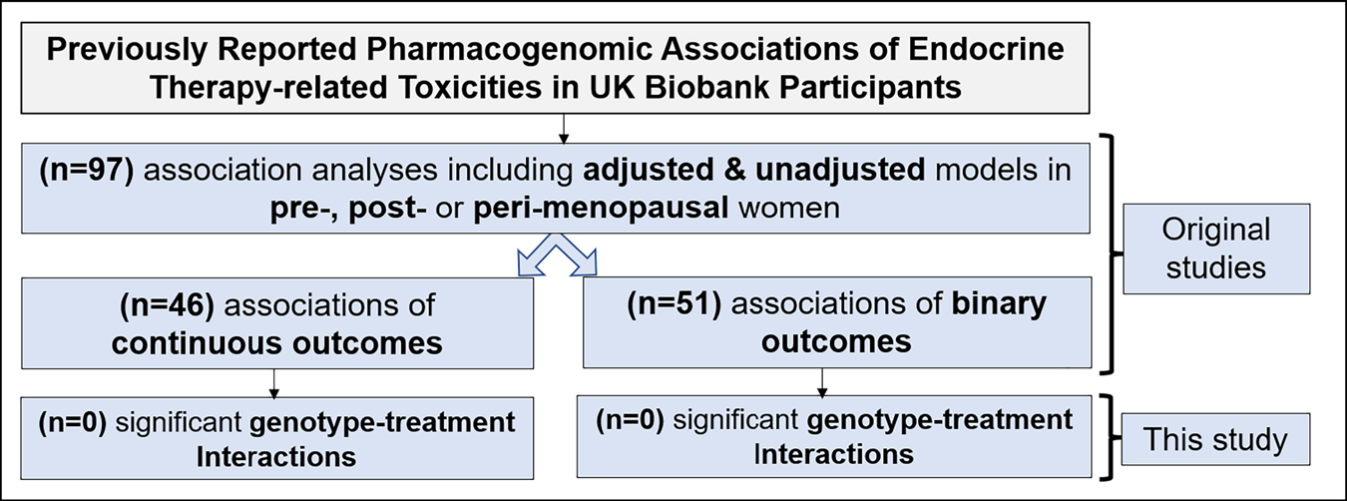
The main results from the UK Biobank analysis of PGx of endocrine therapy-related MIADEs. Associations between previously reported SNVs and MIADEs related to endocrine therapy were assessed in UKBB participants. No statistically significant interactions between treatment and allele status for the risk of MIADEs for any of the variants analysed were observed.

In UKBB, 44% of the variants reported by the investigators of the 24 initial studies were directly genotyped, and 56% were imputed with high confidence (>95%) (Table 4). The initial studies investigating the pharmacogenomics of MIADEs related to endocrine treatment reported 97 statistically significant associations, including 46 associations for continuous outcomes and 51 for binary outcomes (Fig. 2).

**Table 4:** The genomic variants analysed, including frequencies of reference and minor alleles

Gene	SNV ID	Variant type/Consequence	Directly genotyped or imputed	Imputation Score *R*^2^^a^	Chromosome number	Position^b^	allele 1	allele 2	Minor Allele UKBB	MAF UKBB (Unrelated Europeans)
*CYP19A1*	rs10046	3 Prime UTR	Genotyped	N/A	15	51502986	G	A	G	0.47
	rs1008805	Intronic	Imputed	0.990	15	51549599	G	A	G	0.42
	rs1062033	Intronic	Imputed	0.991	15	51547938	C	G	G	0.46
	rs3759811	Intronic	Imputed	1	15	51529265	T	C	T	0.49
	rs4646	3 Prime UTR	Genotyped	N/A	15	51502844	A	C	A	0.26
	rs4775936	Intronic	Imputed	0.996	15	51536022	C	T	T	0.48
	rs2289105	Intronic	Imputed	0.997	15	51507508	T	C	T	0.47
	rs6493497	Upstream	Genotyped	N/A	15	51630835	G	A	A	0.12
	rs700518	Synonymous	Genotyped	N/A	15	51529112	T	C	T	0.49
	rs936308	Intronic	Imputed	0.994	15	51581074	C	G	G	0.14
	rs749292	Intronic	Imputed	0.997	15	51558731	G	A	A	0.45
*CYP11A1*	rs4077581	Promoter	Imputed	1	15	74665514	C	T	C	0.30
	rs11632698	Intronic	Imputed	0.998	15	74637867	A	G	A	0.38
	rs900798	3 Prime UTR	Imputed	0.995	15	74629070	T	G	T	0.31
*CYP2D6*	rs1065852	Missense	Imputed	0.994	22	42526694	G	A	A	0.22
	rs1080985	Upstream	Imputed	0.991	22	42528382	C	G	C	0.23
	rs16947	Missense	Imputed	0.997	22	42523943	A	G	A	0.33
	rs3892097	Splice Acceptor	Imputed	0.992	22	42524947	C	T	T	0.21
	rs28371725	Intronic	Imputed	0.989	22	42523805	C	T	T	0.10
*ESR1*	rs9322335	Intronic	Imputed	0.977	6	152200129	T	C	T	0.26
	rs9340799 *(XbaI)*	Intronic	Genotyped	N/A	6	152163381	A	G	G	0.35
	rs2077647	Synonymous	Genotyped	N/A	6	152129077	T	C	C	0.48
	rs2234693 *(PvuII)*	Intronic	Genotyped	N/A	6	152163335	T	C	C	0.46
	rs2813543	Intronic	Imputed	0.957	6	152424478	A	G	A	0.23
	rs4870061	Intronic	Imputed	0.997	6	152237468	T	C	T	0.25
*ESR2*	rs10140457	Intronic	Imputed	0.994	14	64716693	A	C	C	0.02
	rs4986938	Non-coding	Genotyped	N/A	14	64699816	C	T	T	0.38
*F2*	rs1799963 *(F2 FII G20210A)*	3 Prime UTR	Imputed	0.950	11	46761055	G	A	A	0.01
*F5*	rs6025 *(FVL)*	Missense	Genotyped	N/A	1	169519049	T	C	T	0.02
*CYP17A1*	rs743572	5 Prime UTR	Genotyped	N/A	10	104597152	A	G	G	0.38
*CYP3A4*	rs2740574	Upstream	Genotyped	N/A	7	99382096	C	T	C	0.03
*CYP3A5*	rs776746	Splice Acceptor	Genotyped	N/A	7	99270539	C	T	T	0.07
*TNFRSF11B*	rs2073618	Missense	Genotyped	N/A	8	119964052	G	C	C	0.45
*TNFSF11*	rs7984870	Intronic	Imputed	0.997	13	43146482	G	C	C	0.45
*PTCSC2*	rs10983920	Intronic	Imputed	0.997	9	100602613	C	A	A	0.12
*NCOA1*	rs1804645	Missense	Genotyped	N/A	2	24974958	C	T	T	0.03
*E2F7*	rs310786	Intronic	Genotyped	N/A	12	77436148	C	T	C	0.14
*ABCB1*	rs1045642	Missense	Genotyped	N/A	7	87138645	A	G	G	0.46
*SLC22A23*	rs4959825	Intronic	Imputed	0.988	6	3412240	T	C	T	0.31
*UGT2B7*	rs7439366	Missense	Genotyped	N/A	4	69964338	T	C	C	0.46
*POLQ*	rs9862879	Downstream	Genotyped	N/A	3	121149009	C	T	T	0.12

^a^*R*^2^ is the squared correlation between input genotypes and imputed dosages (i.e. true and inferred genotypes).

^b^Genomic position, build 37 (hg19).

There were a few significant associations in the main effects model. Tamoxifen-treated women carrying the *F5* rs6025 or Factor V Leiden (FVL) variant had increased odds of venous thromboembolism and thromboembolic events under the unadjusted dominant model [OR (95% CI), *P*]: 1.40 (1.18, 1.66), 9.1 × 10^−^^5^ and 1.62 (1.43, 1.83), 5.60 × 10^−^^14^, respectively. However, the genotype‒treatment interaction for these events was not statistically significant: [OR (95% CI), *P*]: 3.02 (1.09, 8.33), 0.033 and 1.95 (0.78, 4.88), 0.15, respectively. In the main effects model, women with prothrombotic mutations (*F5* rs6025 or *F2* rs1799963) also showed increased odds of venous thromboembolism (DVT/PE) in the main effects model [OR (95% CI), *P*]: 1.54 (1.35, 1.77), 4.10 × 10^−^^10^, but this was not significant in the interaction model [OR (95% CI), *P*]: 2.88 (1.18, 7.03), 0.02. The sensitivity analysis indicated that this effect was driven primarily by *F5* rs6025, as the interaction between *F2* rs1799963 and treatment for DVT/PE was not statistically significant according to either the unadjusted or adjusted models: [OR (95% CI), *P*]: 2.09 (0.46, 9.42), 0.34 and 3.04 (0.65, 14.25), 0.16, respectively.

In the main effects model, postmenopausal women on 3rd Gen AIs carrying the *CYP19A1* rs700518 variant showed a significant association with lower BMD (β, 95% CI, P: −0.003 [−0.005, −0.002], 4.54 × 10^−^^6^), but this association was not statistically significant in the interaction model (β, 95% CI, *P*: 0.005 [-0.011, 0.020], 0.55). Similarly, those with the *CYP19A1* rs10046 variant had lower odds of >5% bone loss [OR (95% CI), *P*: 0.95 (0.93, 0.97), 6.80 × 10^−^^5^], but this was not statistically significant in the interaction model [OR (95% CI), *P*: 0.97 (0.73, 1.28), 0.82]. For the *TNFRSF11B* rs2073618 variant, postmenopausal women taking anastrozole or letrozole had higher odds of osteopenia [OR (95% CI), *P*: 1.07 (1.04, 1.10), 6.70 × 10^−^^7^], but this did not hold in the interaction model [OR (95% CI), *P*: 1.18 (0.86, 1.62), 0.31].

Across all 97 regression analyses, no statistically significant interactions were observed between treatment and allele status for either continuous or binary outcomes (Fig. 2). Results were unchanged when applying the Holm step-down procedure for family-wise error control, with no interaction meeting statistical significance after Holm adjustment. A statistically significant interaction would imply that the association between a genomic variant and MIADEs differs according to endocrine therapy exposure, consistent with treatment-dependent genetic effects. In contrast, the observed main-effect associations showed similar effect sizes irrespective of endocrine therapy use, indicating no evidence of treatment-specific modification. All association analyses are detailed in Supplementary Tables S2 and S3.

### Power to detect genotype-treatment interaction analyses

Post hoc power analyses indicated that the study was well powered to detect very large genotype-treatment interaction effects for the rare variants and uncommon adverse outcomes examined; For thromboembolic events, power to detect interaction ORs of five-fold or greater was very high for *F5* rs6025 (97.6% for OR = 5), whereas power to detect five-fold interactions for the lower-frequency *F2* rs1799963 variant was moderate (70%) under Bonferroni correction. Power to detect interaction effects of approximately two- to three-fold magnitude was substantially lower for both variants under stringent multiple-testing correction. For *CYP3A4* rs2740574 and endometrial cancer, power was moderate (73%) for five-fold interaction effects and very limited for three-fold effects (13%), due to the rarity of both the outcome and the variant. In the common-variant sensitivity assessment, power to detect a moderate interaction (OR = 1.5) varied by endpoint after multiple-testing correction; For DVT/PE with *ESR1* rs9340799 (MAF = 0.35), power at the Bonferroni threshold was low for OR = 1.5 (19.02%) but high for OR = 2.0 (93.59%) and effectively complete for OR ≥ 3. For depression with *UGT2B7* rs7439366 (MAF = 0.46), power at Bonferroni was moderate for OR = 1.5 (53.90%) and essentially complete for OR ≥ 2.0 (99.96% for OR = 2.0). Power estimates across variants and assumed interaction effect sizes are summarised in Supplementary Table S4.

## Discussion

Previous pharmacogenomic research has shown that genomic variants may affect toxicity outcomes in breast cancer patients receiving endocrine treatment^13–36^. However, methodological limitations, small sample sizes and conflicting findings underscore the importance of replicating these findings in large, independent cohorts to ensure robustness. In this extensive investigation of a large population cohort, none of the previously reported associations were replicated for either continuous or binary outcomes. Importantly, the absence of statistically significant genotype-treatment interactions does not imply an absence of genetic contribution to MIADEs. Rather, our findings indicate that some alleles may act as general risk factors for these outcomes irrespective of endocrine therapy exposure. Several variants demonstrated significant main-effect associations, supporting their potential relevance for overall risk prediction and monitoring; however, current evidence from our analyses does not support genotype-guided selection between tamoxifen and AIs specifically to reduce MIADE risk.

Although this study represents one of the largest replication efforts to date in this context and is sufficiently powered to exclude very large treatment-specific interaction effects, it cannot rule out more modest but potentially clinically relevant interactions for low-frequency alleles and uncommon adverse events. Post hoc power analyses indicate that, under our stringent multiple-testing correction, the study is primarily powered to detect very large interaction effects. More moderate interaction effects of approximately two- to three-fold magnitude may remain undetected for low-frequency variants and rare MIADEs, even in a cohort of this size, including thromboembolic events associated with *F2* rs1799963 and *F5* rs6025, and endometrial cancer associated with *CYP3A4* rs2740574. Reliable estimation of such effects would require substantially larger or pooled datasets with larger cohorts or alternative study designs with greater numbers of MIADEs. Nevertheless, despite limited power for rare variants and uncommon MIADEs, this study remains substantially larger than most previously published pharmacogenetic studies in this area^9^. Together, these power analyses suggest that limited power is a plausible explanation mainly for modest interaction effects in settings with fewer events and/or lower allele frequencies (Table S4). In particular, for DVT/PE the study had limited power to detect an interaction of OR = 1.5 after Bonferroni correction, so smaller effects cannot be excluded. However, for common variants and sufficiently frequent outcomes, larger interaction effects (OR = 2) very highly powered and for very large interaction effects (OR ≥ 3) would almost certainly have been detected even under stringent correction, and for depression with *UGT2B7* rs7439366, power was essentially complete for OR ≥ 2.0. This reduces the likelihood that non-replication of large previously reported interactions is explained solely by sample size, and instead supports the interpretation that true interaction effects, if present, are likely smaller and/or context dependent.

Our findings contradict those reported in the initial studies, underscoring the importance of conducting large-scale and independent cohort replication prior to considering pharmacogenomic variants in clinical practice. Our findings are, however, consistent with PharmGKB’s current low evidence level (Level 3) assigned to these associations, reflecting the lack of consistent replication across studies. Notably, many previous pharmacogenomic investigations failed to identify significant associations between the variants and endocrine therapy-related ADEs despite the large number of tests performed and in spite of examining multiple variants and several toxicity endpoints^37–68^, suggesting potential false-positive findings. However, comparison with previous pharmacogenomic studies requires careful interpretation. For example, analyses from the BIG 1–98 trial were conducted within a controlled clinical trial setting in postmenopausal women randomised to tamoxifen or letrozole and relied on tumour-derived DNA, enabling assessment of differential genotype effects between endocrine therapy agents (tamoxifen vs. letrozole)^15^. In contrast, our UKBB analysis uses germline genotypes and includes women across a broader age and menopausal spectrum, reflecting real-world endocrine therapy exposure. Importantly, our genotype-treatment interaction evaluates whether genetic associations with MIADEs differ between women exposed to contrasting tamoxifen or AIs and those not exposed, rather than directly contrasting tamoxifen with AIs among treated patients only. Consequently, the two approaches address related but distinct questions, and the lack of statistically significant interaction in our analysis should not therefore be interpreted as contradicting the BIG 1–98 trial findings, but rather as complementary evidence from a population-based setting.

Potential implications for practice and research include the importance of exercising caution when interpreting findings from pharmacogenomic studies and the necessity for adherence to rigorous methodological practices. In contrast to this study, most initial studies failed to consider genotype‒treatment interactions in their analyses, which were incorporated into our regression models to minimise bias. Only 12.5% of the initial studies appropriately incorporated statistical interactions in their analyses. In contrast to this study, which carefully adjusted for covariates and applied Bonferroni correction to manage multiplicity, some earlier studies may not have consistently incorporated these adjustments, potentially increasing the risk of false positives, resulting in less robust conclusions^69^. Taken together, these methodological differences may help explain discrepancies between our results and previously reported associations.

While the study performed an extensive analysis using a large cohort with longitudinal data that is significantly longer than the initial studies and adhered to rigorous methodological practices, it is important to acknowledge a few limitations. First, the phenotypic data within the UKBB, especially those self-reported by participants at baseline, might vary in reliability and quality, which could pose challenges in accurately identifying individuals with relevant conditions^70^. Second, since this analysis included UKBB participants with European ancestry, genetic ancestry findings from this study are not applicable to non-Caucasian populations or broader racial and ethnic groups. Third, participants within the UKBB cohort who carry risk variants might be generally healthier than carriers in the general population, potentially attenuating the pharmacogenomic effects^71^. However, our findings are less confounded compared to those from traditional clinical trials since UKBB participants were not informed about their possession of any specific genetic variant. Finally, while stringent control for multiple testing is essential to minimise Type I error, it can increase the risk of Type II error by reducing sensitivity to detect true but modest effects. In the present study, results were unchanged when applying the Holm step-down procedure for family-wise error control, indicating that the absence of statistically significant genotype-treatment interactions was robust to alternative correction approaches. Detection of interaction effects may be further challenged in large, heterogeneous cohorts, particularly for low-frequency alleles and uncommon adverse outcomes. Accordingly, smaller, more targeted studies may sometimes detect specific genotype-drug-toxicity associations that are difficult to identify in population-based datasets such as UKBB.

In conclusion, this study represents one of the largest replication efforts to date evaluating previously reported pharmacogenomic associations with MIADEs in women receiving endocrine therapy for breast cancer. We found no robust evidence of genotype-treatment interactions after correction for multiple testing. Power analyses indicate that the study was sufficiently powered to exclude very large treatment-specific interaction effects but had limited ability to detect smaller, potentially clinically relevant, interactions for low-frequency alleles and uncommon MIADEs, which would require substantially larger or pooled studies to estimate reliably. At present, the available evidence does not support the use of pharmacogenomic testing to guide individualised endocrine therapy selection in routine clinical practice. Future studies incorporating substantially larger sample sizes and extended follow-up will be required to identify and validate both genomic and non-genomic predictors of endocrine therapy-related adverse outcomes.

## Methods

### Description of study population

UKBB is a large population-based cohort that recruited over 500,000 participants from the general population across England, Scotland and Wales between 2006 and 2010^72^. At enrolment, participants attended one of the assessment centres where they provided blood samples for genomic and biomarker analyses alongside detailed health and lifestyle information. The participants were followed up after the baseline assessment, and their health records information was updated regularly. Longitudinal follow-up has been achieved through repeated self-reported health data and linkage to routinely collected healthcare records up to February 2022 for England and Scotland, and February 2018 for Wales.

As the available samples of other ancestries in the UKBB were insufficient in size to draw any reliable conclusions, we limited our analyses to 389,805 unrelated individuals with genetically determined European ancestry using principal component analysis of genomic data, which is distinct from sociopolitical constructs of ethnicity or race. This aligns with the study’s focus on genetic ancestry as a biological construct relevant to pharmacogenomic association analyses. We included female participants, defined as individuals who were genetically female and self-reported as female, who self-reported taking endocrine agents at the baseline assessment. UKBB does not systematically capture tumour stage, nodal status or recurrence information for all participants. However, in UK clinical practice, endocrine therapy is prescribed in the adjuvant setting for early or localised HR+ breast cancer. We therefore interpret this treated cohort as mainly representing women receiving endocrine therapy for early-stage disease, while acknowledging that a minority of these participants may have received endocrine therapy in the context of advanced or recurrent breast cancer.

### Ascertainment of endocrine therapy exposure

Endocrine therapy exposure was determined using self-reported medication data collected at the baseline assessment. Participants reporting use of relevant medications were identified using UKBB data field 20003. Medications corresponding to tamoxifen and 3rd generation AIs (letrozole, anastrozole, exemestane) were identified using predefined codes. The specific medication codes included in the analysis are detailed in Supplementary Table S5.

### Definition of medically important adverse drug effects (MIADEs)

To address heterogeneity in the terminology and classification of ADEs across the literature, we introduced the term MIADEs to harmonise reporting and improve comparability across different studies. In this study, we defined MIADEs as adverse events that are either serious or severe according to established international frameworks. Serious events were defined according to World Health Organization criteria, including events that are life-threatening, require hospitalisation or result in significant morbidity^73,74^. Severe events correspond to grade 3 to 5 adverse events as defined by the Common Terminology Criteria for Adverse Events (CTCAE)^75^. In addition, MIADEs include events recognised as designated or important medical events by the European Medicines Agency^76,77^. Examples include fractures, venous thromboembolism and second malignancies. Full phenotype definitions, data field identifiers and codes used to define baseline characteristics and MIADE phenotypes are provided in Supplementary Tables S6 and S7.

### Timing and ascertainment of adverse events

Endocrine therapy use was recorded at the baseline assessment, which was therefore used as the index date for adverse outcomes ascertainment. Adverse events recorded prior to baseline were treated as prevalent and were not included as incident outcomes. Consequently, only adverse events occurring after initiation of endocrine therapy were considered incident in the analyses. A fixed latency exclusion window (e.g. excluding events occurring within 1 to 3 months after initiation of endocrine therapy) was not applied, as precise endocrine therapy start dates are not always available with sufficient temporal resolution in UKBB medication self-report data. Inclusion of very early post-initiation events may therefore introduce non-differential misclassification with respect to causal attribution, potentially reducing precision and power to detect genotype-treatment interactions.

Incident MIADEs were ascertained longitudinally using multiple data sources, including Hospital Episode Statistics (HES) inpatient records coded according to ICD-9 and ICD-10 classifications, self-reported health outcomes collected at baseline and during follow-up, and linkages to national death and cancer registries^72^. Together, these linked data enabled longitudinal identification of MIADEs occurring after initiation of endocrine therapy. In alignment with some of the initial studies that stratified analyses by menopausal status, we determined menopausal status in our study to ensure replication of their findings. Menopausal status at baseline in the UKBB was determined by self-reported menstrual history: premenopausal women reported regular menses, postmenopausal women had undergone natural or surgical menopause (e.g. hysterectomy or bilateral oophorectomy), as we previously described^78^. This yielded three categories: premenopausal, postmenopausal and undefined menopause status. All the data and outcomes are sex specific, as the study population consisted solely of female participants. Thus, sex-based comparisons were not applicable, and outcomes were not assessed for sex-specific differences.

### Selection of genomic variants

To identify genomic variants associated with MIADEs related to endocrine treatment, we curated these variants from our recently published systematic review^9^, which involved a comprehensive search across MEDLINE, Embase, Cochrane CENTRAL, Google Scholar and the Pharmacogenomics Knowledge Base (PharmGKB)^79^. We included only variants that showed a statistically significant association with endocrine treatment-related MIADEs.

### Genotyping procedures

The SNP-genotyping array data and imputation used in this study were generated by the UKBB as previously described^12^. Stringent quality control (QC) procedures were applied by the UKBB team to the dataset^80^. We applied additional QC by including only directly genotyped variants that passed QC or imputed variants with an imputation quality >0.95.

### Data analysis and statistical methods

We used linear regression to examine associations between baseline measurements and genetic variants, and logistic regression for associations between incident MIADE phenotypes and genetic variants. This involved two distinct analyses: one for continuous outcomes using baseline data, and another for binary outcomes using baseline data, follow-up visits, and updated HES data.

Regression analyses were conducted using both main-effect and interaction models. Main-effect models were used to assess associations between genotype and MIADEs by comparing individuals with and without a given allele, with multivariable adjustment for treatment exposure and other potential confounders. In interaction models, the genotype × treatment interaction tests whether the association between genotype and MIADEs differs between women exposed to endocrine therapy and those not exposed; This interaction therefore evaluates treatment exposure-dependent genetic effects, rather than directly contrasting specific endocrine agents such as tamoxifen versus AIs among treated patients only.

Variants were analysed using dominant, recessive, or additive genetic models, as specified in the initial studies. Statistical analyses were adjusted for the assessment centre and the first five genetic principal components to account for population stratification and potential confounders, as reported in the initial studies. Bonferroni correction was used to account for multiple testing, resulting in a critical *p*-value of 5.15 × 10^−4^ (0.05 corrected for 97 tests: *α* = 0.05/97 = 5.15 × 10^−4^). STATA (version 16.0) and R were used for statistical analysis, and the analyses were two-tailed. As a sensitivity analysis, the Holm step-down procedure for family-wise error control^81^ was also applied to the genotype-treatment interaction tests to assess the robustness of results to alternative multiple-testing correction.

### Power assessment

Power considerations are particularly important for genotype-treatment interaction analyses involving rare variants and uncommon adverse outcomes^82^. Thus, to evaluate whether the absence of statistically significant genotype-treatment interactions could plausibly reflect limited statistical power, we conducted post hoc power analyses for interaction tests involving rare variants and uncommon adverse outcomes for interaction odds ratios (ORs) of three-, five- and ten-fold magnitude. In addition, because most curated variants were common in UKBB as 36/41 variants had minor allele frequencies (MAF) ≥ 0.05, we repeated post hoc power calculations for representative common variants and more frequent outcomes. Power was assessed using the observed numbers of treated and non-treated women, adverse event counts in treated and non-treated women and MAF in UKBB. Power was calculated for assumed interaction effects (ORs) of 1.5, 2, 3, 5 and 10. Power was calculated under a conventional two-sided significance threshold *α* = 0.05 and the Bonferroni-corrected threshold (*α* = 5.15 × 10^−4^). This approach follows standard information-based power calculation methods for logistic regression and interaction terms^83–85^.

### Ethics and consent

UK Biobank has ethical approval as a Research Tissue Bank from the North West—Haydock Research Ethics Committee (REC reference 11/NW/0382; RTB renewals 16/NW/0274 and 21/NW/0157)(https://www.ukbiobank.ac.uk/about-us/how-we-work/ethics/). All procedures were performed in accordance with the Declaration of Helsinki. All participants provided written informed consent at enrolment. This study was conducted under UK Biobank application numbers 49847 and 9072.

## Supplementary information

Supplementary information

## Data Availability

The genetic and phenotypic data from the UK Biobank can be accessed by applying through their website (www.ukbiobank.ac.uk/register-apply). We cannot directly grant access to the specific data fields used in this study. All other data relevant to the study are included in the article or uploaded as supplementary information.
