# Supplementary material for: No evidence for genotype-treatment interactions with breast cancer endocrine therapy adverse effects in UK Biobank
Source: NPJ Breast Cancer. 2026 Feb 26;12:53. doi: 10.1038/s41523-026-00923-2 (PMC13062014; doi:10.1038/s41523-026-00923-2)
Supplement: Supplementary file 1 — Supplementary information [file 41523_2026_923_MOESM1_ESM.pdf]

Supplementary Materials

Table S1 *Status of including interaction effects in analytical models conducted by authors of pharmacogenetic studies on MIADEs related to endocrine therapy.*

Study [Author, Year]	The interaction effects model was used appropriately*
Al-Mamun 2017	No
Argalacsova 2017	No
Baatjes 2020	No
Chu 2007	No
Dieudonné 2014	No
Garber 2010	No
Hartmaier 2012	No
Koukouras 2012	No
Kovac 2015	No
Leyland-Jones 2015 (1)	Yes
Leyland-Jones 2015 (2)	Yes
Mazzuca 2016	No
Miranda 2021	No
Napoli 2013	No
Ntukidem 2008	No
Oesterreich 2015	No
Ohnishi 2005	No
Onitilo 2009	No
Rodríguez-Sanz 2015	No
Santa-Maria 2016	Yes
Wang 2013	No
Wang 2015	No
Weng 2013	No
Wickramage 2017	No

**The frequency of the failure to consider potential interaction effects in the analyses was assessed using the following search terms: moderator, heterogeneity of effect, magnifier, qualifier, synergy, buffering effect, statistical interactions, effect modification, effect modifier, multiplicative or synergistic.*

Table S2 *Main-effect and treatment-genotype interaction analyses for continuous MIADE outcomes in the UK Biobank*

System Organ Class	Regimen or Drug(s)	Gene	SNP rs# ID	Genotype	Statistical genetic model	Toxicity endpoint or Parameter	Menopausal status	Original study			Replication attempt in UK Biobank					
								Original study	Adjustement	Effect size	Adjustment	Statistical model	Effect size	lC95%	UC95%	p_value
**Musculoskeletal Disorders**	**Anastrozole; Exemestane; Letrozole**	***CYP19A1***	**rs700518**	**A**	**Recessive**	**Bone mineral density**	**Post-menopausal**	**Napoli 2013**	**BMI and baseline BMD**	**Means % Change BMD with 1 y± SD: AA vs. AG+GG [-2.684± 2.34 vs. -0.4± 2.11], (P = 0.03)**	**BMI, assessment centre, first five principal components**	**Main effects model**	**β coefficient=-0.003**	**-0.005**	**-0.002**	**4.54E-06**
												Interaction model	β coefficient=0.00477239	-0.010756	0.0203008	5.47E-01
Musculoskeletal Disorders	Tamoxifen	*NCOA1*	rs1804645	T	Additive	Bone mineral density	Pre- , Peri- & Post-menopausal	Hartmaier 2012	N/R (individuals receiving chemotherapy were excluded from analysis)	Mean % Change BMD with 1 yr of tamoxifen therapy in the lumbar (WT:−1.30% ± 0.55, n=107; SNP:−6.43% ± 1.62, n=4; P=0.037)	Assessment centre, first five principal components [those receiving chemotherapy were excluded]	Main effects model	β coefficient=-0.00120996	-0.0035125	0.0010925	3.03E-01
												Interaction model	β coefficient=0.00848767	-0.0309076	0.047883	6.73E-01
Musculoskeletal Disorders	Letrozole; Exemestane	*CYP19A1*	rs6493497	A	Recessive	T-score	Post-menopausal	Oesterreich 2015	Age, BMI, prior chemotherapy or tamoxifen	Mean Change T score (SD)= VT/VT −2.90 (0) vs. WT/WT, WT/VT −0.37 (0.44), p=4.6E−8	Age, BMI, prior chemotherapy, assessment centre, first five principal components	Main effects model	β coefficient=-0.0119326	-0.063995	0.0401298	6.50E-01
												Interaction model	β coefficient=-0.4482673	-1.630686	0.7341511	4.60E-01
Musculoskeletal Disorders	Letrozole; Exemestane	*ESR1*	rs4870061	T	Recessive	T-score	Post-menopausal	Oesterreich 2015	Age, BMI, prior chemotherapy or tamoxifen	Mean Change T score (SD)= VT/VT −1.07 (1.01) vs. WT/WT, WT/VT −0.34 (0.40), p=3.2E−7	Age, BMI, prior chemotherapy, assessment centre, first five principal components	Main effects model	β coefficient=-0.0138345	-0.0383734	0.0107045	2.70E-01
												Interaction model	β coefficient=0.1409758	-0.2913455	0.5732971	5.20E-01
Musculoskeletal Disorders	Letrozole; Exemestane	*ESR1*	rs4870061	T	Recessive	T-score	Post-menopausal	Oesterreich 2015	Age, BMI, prior chemotherapy or tamoxifen	Mean Change T score (SD)=WT/VT −0.39 (0.40) vs. VT/VT 1.07 (1.01) vs. WT/WT −0.31 (0.40), p=5.2E−5	Age, BMI, prior chemotherapy, assessment centre, first five principal components	Main effects model	β coefficient=-0.0089843	-0.0187114	0.0007429	7.00E-02
												Interaction model	β coefficient=0.1186838	-0.0598134	0.297181	1.90E-01
Musculoskeletal Disorders	Letrozole; Exemestane	*ESR1*	rs9322335	T	Recessive	T-score	Post-menopausal	Oesterreich 2015	Age, BMI, prior chemotherapy or tamoxifen	Mean Change T score (SD)=VT/VT 0.44 (0.63) vs. WT/WT, WT/VT −0.29 (0.47), p=3.5E−5	Age, BMI, prior chemotherapy, assessment centre, first five principal components	Main effects model	β coefficient=-0.0031943	-0.0271939	0.0208054	7.90E-01

												Interaction model	β coefficient=0.4719414	0.0120968	0.931786	4.40E-02
Musculoskeletal Disorders	Letrozole	*ESR1*	rs4870061	T	Recessive	T-score	Post-menopausal	Oesterreich 2015	Age, BMI, prior chemotherapy or tamoxifen	Mean Change T score (SD)=VT/VT −1.25 (0.90) vs. WT/WT, WT/VT −0.36 (0.39), p=3.1E–6	Age, BMI, prior chemotherapy, assessment centre, first five principal components	Main effects model	β coefficient=-0.0138436	-0.0383835	0.0106962	2.70E-01
												Interaction model	β coefficient=0.1247933	-0.4244687	0.6740553	6.60E-01
Musculoskeletal Disorders	Letrozole	*ESR1*	rs9322335	T	Recessive	T-score	Post-menopausal	Oesterreich 2015	Age, BMI, prior chemotherapy or tamoxifen	Mean Change T score (SD)=VT/VT 0.54 (0.63) vs. WT/WT, WT/VT −0.35 (0.40), p=5.2E–6	Age, BMI, prior chemotherapy, assessment centre, first five principal components	Main effects model	β coefficient=-0.0031724	-0.0271728	0.0208281	8.00E-01
												Interaction model	β coefficient=0.5900829	-0.0185096	1.198675	5.70E-02
Musculoskeletal Disorders	Exemestane	*CYP19A1*	rs6493497	A	Recessive	T-score	Post-menopausal	Oesterreich 2015	Age, BMI, prior chemotherapy or tamoxifen	Mean Change T score (SD)=VT/VT −2.90 (0) vs. WT/WT, WT/VT −0.30 (0.38), p=1.6E–9	Age, BMI, prior chemotherapy, assessment centre, first five principal components	Main effects model	β coefficient=-0.0115203	-0.0635841	0.0405434	6.60E-01
												Interaction model	β coefficient=-0.4287698	-1.617906	0.7603667	4.80E-01
Musculoskeletal Disorders	Exemestane	*ESR1*	rs2813543	A	Recessive	T-score	Post-menopausal	Oesterreich 2015	Age, BMI, prior chemotherapy or tamoxifen	Mean Change T score (SD)=VT/VT −1.55 (1.91) vs. WT/WT, WT/VT −0.31 (0.41), p=2.1E–4	Age, BMI, prior chemotherapy, assessment centre, first five principal components	Main effects model	β coefficient=-0.0057766	-0.032267	0.0207137	6.70E-01
												Interaction model	β coefficient=-0.8836432	-2.33382	0.566534	2.30E-01
Musculoskeletal Disorders	Letrozole	*ESR1*	rs4870061	T	Recessive	Bone mineral density	Post-menopausal	Oesterreich 2015	Age, BMI, prior chemotherapy or tamoxifen	Mean % Change BMD (SD)= VT/VT −10.94 (12.71) vs. WT/WT, WT/VT −3.76 (3.49), p=3.0E–4	Age, BMI, prior chemotherapy, assessment centre, first five principal components	Main effects model	β coefficient=-0.001866	-0.0044691	0.000737	1.60E-01
												Interaction model	β coefficient=0.0192075	-0.0366608	0.0750759	5.00E-01
Musculoskeletal Disorders	Letrozole	*ESR2*	rs10140457	C	Additive	Bone mineral density	Post-menopausal	Oesterreich 2015	Age, BMI, prior chemotherapy or tamoxifen	Mean % Change BMD (SD)= WT/VT 3.08 (6.92) vs. WT/WT −3.43 (3.97), p=3.0E–4	Age, BMI, prior chemotherapy, assessment centre, first five principal components	Main effects model	β coefficient=-0.0013776	-0.0049584	0.0022032	4.50E-01
												Interaction model	β coefficient=-0.0603724	-0.1628594	0.0421146	2.50E-01

Musculoskeletal Disorders	Anastrozole; Exemestane; Letrozole	*CYP11A1*	rs11632698	A	Additive	Bone mineral density	Post-menopausal	Rodríguez-Sanz 2015	Age, BMI, prior chemotherapy or tamoxifen. Patients with scoliosis, on bisphosphonates (BP) treatment, spine scan artifacts and/or those with hip scan artifacts or bilateral prostheses were excluded from the study.	β coefficient (95% CI), P value= 0.94 (0.30–1.58), 0.004	Age, BMI, prior chemotherapy, assessment centre, first five principal components [those with scoliosis, on bisphosphonates (BP) treatment, spine scan artifacts and/or those with hip scan artifacts or bilateral prostheses were excluded]	Main effects model	β coefficient=0.00063477	-0.0002871	0.0015566	1.77E-01
											Interaction model	β coefficient=0.00354739	-0.0074157	0.0145105	5.26E-01
Musculoskeletal Disorders	Anastrozole; Exemestane; Letrozole	*CYP11A1*	rs900798	T	Additive	Bone mineral density	Post-menopausal	Rodríguez-Sanz 2015	Age, BMI, prior chemotherapy or tamoxifen. Patients with scoliosis, on bisphosphonates (BP) treatment, spine scan artifacts and/or those with hip scan artifacts or bilateral prostheses were excluded from the study.	β coefficient (95% CI), P value= 1.06 (0.39–1.72), 0.002	Age, BMI, prior chemotherapy, assessment centre, first five principal components [those with scoliosis, on bisphosphonates (BP) treatment, spine scan artifacts and/or those with hip scan artifacts or bilateral prostheses were excluded]	Main effects model	β coefficient=0.00051465	-0.0004526	0.0014819	2.97E-01
											Interaction model	β coefficient=0.00312909	-0.008436	0.0146942	5.96E-01
Musculoskeletal Disorders	Anastrozole; Exemestane; Letrozole	*CYP11A1*	rs4077581	C	Additive	Bone mineral density	Post-menopausal	Rodríguez-Sanz 2015	Age, BMI, prior chemotherapy or tamoxifen. Patients with scoliosis, on bisphosphonates (BP) treatment, spine scan artifacts and/or those with hip scan artifacts or bilateral prostheses were excluded from the study.	β coefficient (95% CI), P value= 0.98 (0.30–1.66), 0.005	Age, BMI, prior chemotherapy, assessment centre, first five principal components [those with scoliosis, on bisphosphonates (BP) treatment, spine scan artifacts and/or those with hip scan artifacts or bilateral prostheses were excluded]	Main effects model	β coefficient=0.00050966	-0.0004745	0.0014938	3.10E-01
											Interaction model	β coefficient=0.00410373	-0.0076801	0.0158875	4.95E-01
Musculoskeletal Disorders	Anastrozole; Letrozole	*TNFRSF11B*	rs2073618	C	Recessive	Bone mineral density	Post-menopausal	Wang 2015	N/R	Mean ± SD, (n) =CC vs. GG: 0.801 ± 0.218, (n = 50) vs. 0.987 ± 0.257 (n = 59), P value=0.049	Assessment centre, first five principal components	Main effects model	Means ± SD:CC vs. GG. [0.4749674 ±0.1197611 vs.0.4789469 ± 0.1035114], (P=0.6952) Diff in mean=-0.0039795, Std. Err.= 0.0101507, [95% Conf. Interval]= [-0.0239225, 0.0159635], P=0.6952			
											Interaction model	β coefficient=0.00285258	-0.0158902	0.0215954	7.65E-01

Musculoskeletal Disorders	Tamoxifen	*E2F7*	rs310786	C	Additive	Bone mineral density	Pre- , Peri- & Post-menopausal	Weng 2013	Age, menopausal status, prior chemotherapy	Log2 [Lumbar bone loss, (min, max)] CC= 24.4 [-77.43, 90.6] vs. CT= 14.76 [-90.42, 92.8] vs. TT= -1.73 [-120, 129.9] (P = 0.043)	Age, menopausal status, prior chemotherapy, assessment centre, first five principal components	Main effects model	β coefficient=0.0007763	-0.0003125	0.0018651	1.62E-01
												Interaction model	β coefficient=0.00108519	-0.0135412	0.0157116	8.84E-01
Musculoskeletal Disorders	Tamoxifen	*POLQ*	rs9862879	T	Additive	Bone mineral density	Pre- , Peri- & Post-menopausal	Weng 2013	Age, menopausal status, prior chemotherapy	N/R	Age, menopausal status, prior chemotherapy, assessment centre, first five principal components	Main effects model	β coefficient=-0.00076705	-0.0019399	0.0004058	2.00E-01
												Interaction model	β coefficient=-0.00064037	-0.0164943	0.0152136	9.37E-01
Musculoskeletal Disorders	Tamoxifen	*SLC22A23*	rs4959825	T	Additive	Bone mineral density	Pre- , Peri- & Post-menopausal	Weng 2013	Age, menopausal status, prior chemotherapy	N/R	Age, menopausal status, prior chemotherapy, assessment centre, first five principal components	Main effects model	β coefficient=-0.00017619	-0.0009992	0.0006468	6.75E-01
												Interaction model	β coefficient=0.00470449	-0.006717	0.016126	4.19E-01
Musculoskeletal Disorders	Tamoxifen	*E2F7*	rs310786	C	Recessive	Bone mineral density	Pre- , Peri- & Post-menopausal	Weng 2013	Age, menopausal status, prior chemotherapy	Log2 [Lumbar bone loss, (min, max)] CC= 24.4 [-77.43, 90.6] vs. CT= 14.76 [-90.42, 92.8] vs. TT= -1.73 [-120, 129.9] (P = 0.043)	Age, menopausal status, prior chemotherapy, assessment centre, first five principal components	Main effects model	β coefficient=0.00084713	-0.0028745	0.0045687	6.55E-01
												Interaction model	β coefficient=0.01958579	-0.0308838	0.0700554	4.47E-01
Musculoskeletal Disorders	Tamoxifen	*PTCSC2*	rs10983920	A	Additive	Bone mineral density	Pre- , Peri- & Post-menopausal	Weng 2013	Age, menopausal status, prior chemotherapy	N/R	Age, menopausal status, prior chemotherapy, assessment centre, first five principal components	Main effects model	β coefficient=-0.00001606	-0.0011662	0.0011341	9.78E-01
												Interaction model	β coefficient=0.00354694	-0.0118096	0.0189035	6.51E-01
Metabolism Disorders	Anastrozole; Exemestane; Letrozole	*ESR1*	XbaI (rs9340799)	A	Recessive	Triglycerides	Post-menopausal	Koukouras 2012	N/R	XbaI (AA)= 159 ± 42 vs. XbaI (AG+GG)=136 ± 40 [p = 0.044] (mean value ± SD)	Assessment centre, first five principal components	Main effects model	β coefficient=0.000039	-0.009657	0.009735	9.94E-01
Metabolism Disorders	Anastrozole; Exemestane; Letrozole	*ESR1*	XbaI (rs9340799)	A	Recessive	Triglycerides	Post-menopausal	Koukouras 2012	N/R	XbaI (AA)= 159 ± 42 vs. XbaI (AG+GG)=136 ± 40 [p = 0.044] (mean value ± SD)	Assessment centre, first five principal components	Main effects model	XbaI (AA)=1.782029 ± 1.000734 vs. XbaI (AG+GG)= 1.715918 ± .8683153 [p =0.2268] (mean value ± SD), diff in mean=.066111, Std. Err.=.0546713, [95% Conf. Interval]= [-			

													.0411528 .1733748], P=0.2268			
												Interaction model	β coefficient=0.06775764	-0.033992	0.1695073	1.92E-01
Metabolism Disorders	Anastrozole; Exemestane; Letrozole	*ESR1*	PvuII (rs2234693)	C	Recessive	Triglycerides	Post-menopausal	Koukouras 2012	N/R	PvuII (CC)= 161 ± 47 vs. PvuII (TC+TT)= 133 ± 39 [p = 0.015] (mean value ± SD)	Assessment centre, first five principal components	Main effects model	β coefficient=-0.00064186	-0.0124033	0.0111195	9.15E-01
Metabolism Disorders	Anastrozole; Exemestane; Letrozole	*ESR1*	PvuII (rs2234693)	C	Recessive	Triglycerides	Post-menopausal	Koukouras 2012	N/R	PvuII (CC)= 161 ± 47 vs. PvuII (TC+TT)= 133 ± 39 [p = 0.015] (mean value ± SD)	Assessment centre, first five principal components	Main effects model	PvuII (CC)= 1.7171 ± .9579613 vs. PvuII (TC+TT)=1.750289 ± .9171709 [p=0.6154] (mean value ± SD), diff in mean=-.033189, Std. Err.=.0660513, [95% Conf. Interval]= [-.1627803 .0964023], P =0.6154			
												Interaction model	β coefficient=-0.03411345	-0.1569839	0.088757	5.86E-01
Metabolism Disorders	Anastrozole; Exemestane; Letrozole	*ESR1*	XbaI (rs9340799)	A	Recessive	LDL	Post-menopausal	Koukouras 2012	N/R	XbaI (AA)= 157 ± 20 vs. XbaI (AG+GG)= 142 ± 23 [p = 0.025] (mean value ± SD)	Assessment centre, first five principal components	Main effects model	β coefficient=-0.00133949	-0.0110291	0.0083501	7.86E-01
Metabolism Disorders	Anastrozole; Exemestane; Letrozole	*ESR1*	XbaI (rs9340799)	A	Recessive	LDL	Post-menopausal	Koukouras 2012	N/R	XbaI (AA)= 157 ± 20 vs. XbaI (AG+GG)= 142 ± 23 [p = 0.025] (mean value ± SD)	Assessment centre, first five principal components	Main effects model	XbaI (AA)=3.746609 ± .9033583 vs. XbaI (AG+GG)= 3.769003 ± .878145 [p = 0.6700] (mean value ± SD), diff in mean=-.022394, Std. Err.=.0525323, [95% Conf. Interval]= [-.1254613 .0806733], P =0.6700			
												Interaction model	β coefficient=-0.02080825	-0.1224952	0.0808787	6.88E-01
Metabolism Disorders	Anastrozole; Exemestane; Letrozole	*ESR1*	PvuII (rs2234693)	C	Recessive	LDL	Post-menopausal	Koukouras 2012	N/R	PvuII (CC)= 155 ± 20 vs. PvuII (TC+TT)= 141 ± 24 [p = 0.026] (mean value ± SD)	Assessment centre, first five principal components	Main effects model	β coefficient=-0.00120545	-0.0129589	0.010548	8.41E-01
Metabolism Disorders	Anastrozole; Exemestane; Letrozole	*ESR1*	PvuII (rs2234693)	C	Recessive	LDL	Post-menopausal	Koukouras 2012	N/R	PvuII (CC)= 155 ± 20 vs. PvuII (TC+TT)= 141 ± 24 [p = 0.026] (mean value ± SD)	Assessment centre, first five principal components	Main effects model	PvuII (CC)= 3.791319 ± .836355 vs. PvuII (TC+TT)= 3.751296 ± .9019727 [p = 0.5286] (mean value ± SD), diff in mean=.040023, Std. Err.= .0634987, [95% Conf. Interval]= [-.0845602 .1646062], P =0.5286			
												Interaction model	β coefficient=0.04104445	-0.0818835	0.1639724	5.13E-01

Metabolism Disorders	Tamoxifen	*ESR1*	XbaI (rs9340799)	G	Recessive	Total cholesterol	Post-menopausal	Ntukidem 2008	N/R (patients were neither perimenopausal nor were taking concomitant lipid-lowering medications)	[Change in serum lipid particle concentration (mg/dl) with 95% CI, p-values between baseline and 4 months: AA/AG vs the GG alleles. Total cholesterol: AA [−22 (−30, −14)], AG [−20 (−30, −10)], GG [−40 (−58, −22)], a statistically significant difference for the AA/AG versus GG genotypes (P=0.03]	Assessment centre, first five principal components [those taking lipid lowering meds were excluded]	Main effects model	β coefficient=-0.00622678	-0.0249079	0.0124544	5.14E-01
												Interaction model	β coefficient=-0.03504523	-0.2813176	0.2112272	7.80E-01
Metabolism Disorders	Tamoxifen	*ESR1*	XbaI (rs9340799)	G	Additive	Triglycerides	Pre-menopausal	Ntukidem 2008	N/R (patients were neither perimenopausal nor were taking concomitant lipid-lowering medications)	[Change in serum lipid particle concentration (mg/dl) with 95% CI, p-values between baseline and 4 months: (−) 7mg/dl, (+) 46mg/dl, and (+) 163 mg/dl for women who carry the AA, AG, and GG genotypes, respectively (P=0.002)	Assessment centre, first five principal components [those taking lipid lowering meds were excluded]	Main effects model	β coefficient=0.0044995	-0.0047509	0.0137499	3.40E-01
												Interaction model	β coefficient=-0.0383777	-0.1764855	0.0997302	5.90E-01
Metabolism Disorders	Tamoxifen	*ESR1*	XbaI (rs9340799)	G	Additive	HDL cholesterol	Pre-menopausal	Ntukidem 2008	N/R (patients were neither perimenopausal nor were taking concomitant lipid-lowering medications)	[Change in serum lipid particle concentration (mg/dl) with 95% CI, p-values between baseline and 4 months: (+) 5mg/dl, (−) 0.6mg/dl, and (−) 4 mg/dl for the AA, AG, and GG genotypes, respectively (P=0.004 for gene–dose effect).	Assessment centre, first five principal components [those taking lipid lowering meds were excluded]	Main effects model	β coefficient=-0.0031129	-0.0077855	0.0015598	1.90E-01
												Interaction model	β coefficient=-0.0063933	-0.0779233	0.0651367	8.60E-01

Metabolism Disorders	Tamoxifen	*ESR2*	ESR2-02 (rs4986938)	A	Additive	Triglycerides	Pre-menopausal	Ntukidem 2008	N/R (patients were neither perimenopausal nor were taking concomitant lipid-lowering medications)	[Change in serum lipid particle concentration (mg/dl) with 95% CI, p-values between baseline and 4 months: (+) 97 mg/dl, (+) 37 mg/dl, and (+) 7 mg/dl for the GG, AG, and AA genotypes in ESR2-02, respectively (P=0.04 for gene–dose effect)	Assessment centre, first five principal components [those taking lipid lowering meds were excluded]	Main effects model	β coefficient=0.003462	-0.0056922	0.0126163	4.60E-01
												Interaction model	β coefficient=0.0941782	-0.0509734	0.2393298	2.00E-01
Metabolism Disorders	Tamoxifen	*ESR2*	ESR2-02 (rs4986938)	A	Additive	Triglycerides	Post-menopausal	Ntukidem 2008	N/R (patients were neither perimenopausal nor were taking concomitant lipid-lowering medications)	[Change in serum lipid particle concentration (mg/dl) with 95% CI, p-values between baseline and 4 months: (+) 71 mg/dl, (+) 8 mg/dl, and (−) 15 mg/dl from baseline for the GG, AG, and AA genotypes in ESR2-02, respectively (P=0.01 for gene—dose effect)	Assessment centre, first five principal components [those taking lipid lowering meds were excluded]	Main effects model	β coefficient=-0.00215755	-0.0091482	0.0048331	5.45E-01
												Interaction model	β coefficient=-0.02455295	-0.1191859	0.07008	6.11E-01
Metabolism Disorders	Letrozole	*CYP19A1*	rs10046	A	Recessive	Triglycerides	Post-menopausal	Santa-Maria 2016	Age, BMI, race, change in plasma estradiol, and prior hormonal therapy and tamoxifen use	Mean Change, mg/dL (SE), P-value= −36.4 (7.8), 0.0000069. Only Caucasian women: -30.92 (8.2), 0.00023. Without adjusting for changes in plasma estradiol concentrations: -29.8 (7.3), 0.000082	Age, BMI, HRT, assessment centre, first five principal components	Main effects model	β coefficient=-0.0069599	-0.0171134	0.0031936	1.80E-01
												Interaction model	β coefficient=0.1383645	-0.0827408	0.3594697	2.20E-01

Metabolism Disorders	Letrozole	*CYP19A1*	rs2289105	C	Recessive	Triglycerides	Post-menopausal	Santa-Maria 2016	Age, BMI, race, change in plasma estradiol, and prior hormonal therapy and tamoxifen use	Mean Change, mg/dL (SE), P-value= −36.45 (7.8), 0.0000074. Only Caucasian women: -30.98 (8.2), 0.00024. Without adjusting for changes in plasma estradiol concentrations: -29.8 (7.4), 0.000085	Age, BMI, HRT, assessment centre, first five principal components	Main effects model	β coefficient=-0.0072335	-0.0173706	0.0029035	1.60E-01
												Interaction model	β coefficient=0.1322193	-0.0882891	0.3527277	2.40E-01
Metabolism Disorders	Letrozole	*CYP19A1*	rs3759811	C	Recessive	Triglycerides	Post-menopausal	Santa-Maria 2016	Age, BMI, race, change in plasma estradiol, and prior hormonal therapy and tamoxifen use	Mean Change, mg/dL (SE), P-value= −32.3 (9.0), 0.00045. Only Caucasian women: -33 (9.4) 0.00065. Without adjusting for changes in plasma estradiol concentrations: -30.8 (8.5), 0.00043	Age, BMI, HRT, assessment centre, first five principal components	Main effects model	β coefficient=-0.0076403	-0.0180249	0.0027442	1.50E-01
												Interaction model	β coefficient=0.1019051	-0.127324	0.3311343	3.80E-01
Metabolism Disorders	Letrozole	*CYP19A1*	rs700518	G	Recessive	Triglycerides	Post-menopausal	Santa-Maria 2016	Age, BMI, race, change in plasma estradiol, and prior hormonal therapy and tamoxifen use	Mean Change, mg/dL (SE), P-value= −32.3 (8.4), 0.00019	Age, BMI, HRT, assessment centre, first five principal components	Main effects model	β coefficient=-0.0075438	-0.0179292	0.0028417	1.50E-01
												Interaction model	β coefficient=0.1018087	-0.1274206	0.3310379	3.80E-01
Metabolism Disorders	Letrozole	*CYP19A1*	rs4775936	C	Recessive	Triglycerides	Post-menopausal	Santa-Maria 2016	Age, BMI, race, change in plasma estradiol, and prior hormonal therapy and tamoxifen use	Mean Change, mg/dL (SE), P-value= −33.2 (8.9), 0.00028	Age, BMI, HRT, assessment centre, first five principal components	Main effects model	β coefficient=0.0084503	-0.0018386	0.0187391	1.10E-01
												Interaction model	β coefficient=-0.0955153	-0.3611857	0.1701552	4.80E-01
Metabolism Disorders	Letrozole	*CYP19A1*	rs4646	A	Additive	HDL cholesterol	Post-menopausal	Santa-Maria 2016	Age, BMI, race, change in plasma estradiol, and prior hormonal therapy and tamoxifen use	Mean Change, mg/dL (SE), P-value= −4.2 (1.16), 0.00043	Age, BMI, HRT, assessment centre, first five principal components	Main effects model	β coefficient=-0.0033432	-0.00667	-0.0000165	4.90E-02
												Interaction model	β coefficient=-0.0321437	-0.1167246	0.0524372	4.60E-01

Metabolism Disorders	Letrozole	*CYP19A1*	rs749292	A	Recessive	Triglycerides	Post-menopausal	Santa-Maria 2016	Age, BMI, race, change in plasma estradiol, and prior hormonal therapy and tamoxifen use	Mean Change, mg/dL (SE), P-value= −39.3 (8.6), 0.000012. Only Caucasian women: -37.47 (9.3), 0.000095. Without adjusting for changes in plasma estradiol concentrations: -32.8 (8.3), 0.00012	Age, BMI, HRT, assessment centre, first five principal components	Main effects model	β coefficient=-0.007234	-0.0187133	0.0042453	2.20E-01
												Interaction model	β coefficient=0.1593088	-0.1022963	0.4209139	2.30E-01
Metabolism Disorders	Letrozole	*CYP19A1*	rs749292	A	Additive	Triglycerides	Post-menopausal	Santa-Maria 2016	Age, BMI, race, change in plasma estradiol, and prior hormonal therapy and tamoxifen use	Mean Change, mg/dL (SE), P-value= −20.2 (4.7), 0.000037. Only Caucasian women: -19.51 (5.0), 0.00017. Without adjusting for changes in plasma estradiol concentrations: -17.1 (4.5), 0.00021	Age, BMI, HRT, assessment centre, first five principal components	Main effects model	β coefficient=-0.005955	-0.0124772	0.0005672	7.40E-02
												Interaction model	β coefficient=0.092874	-0.068907	0.254655	2.60E-01
Metabolism Disorders	Letrlipid [taking letrozole and on lipid-altering medications]	*CYP19A1*	rs749292	A	Additive	HDL cholesterol	Post-menopausal	Santa-Maria 2016	Age, BMI, race, change in plasma estradiol, and prior hormonal therapy and tamoxifen use	Mean Change, mg/dL (SE), P-value= 6.7 (1.4), 0.000037. Only Caucasian women: 6 (1.3), 0.000065.	Age, BMI, HRT, assessment centre, first five principal components	Main effects model	β coefficient=0.0016827	-0.0012467	0.0046122	2.60E-01
												Interaction model	β coefficient=0.0155252	-0.1687571	0.1998074	8.70E-01
Metabolism Disorders	Letrlipid [taking letrozole and on lipid-altering medications]	*CYP19A1*	rs1062033	G	Additive	HDL cholesterol	Post-menopausal	Santa-Maria 2016	Age, BMI, race, change in plasma estradiol, and prior hormonal therapy and tamoxifen use	Mean Change, mg/dL (SE), P-value= 6.9 (1.5), 0.000043. Only Caucasian women: 6.2 (1.3), 0.000023	Age, BMI, HRT, assessment centre, first five principal components	Main effects model	β coefficient=0.0023691	-0.0005492	0.0052873	1.10E-01
												Interaction model	β coefficient=0.0132003	-0.1710833	0.1974839	8.90E-01
Metabolism Disorders	Letrlipid [taking letrozole and on lipid-altering medications]	*CYP19A1*	rs1062033	G	Dominant	HDL cholesterol	Post-menopausal	Santa-Maria 2016	Age, BMI, race, change in plasma estradiol, and prior hormonal therapy and tamoxifen use	Mean Change, mg/dL (SE), P-value= Only Caucasian women: 8.26, 0.00035	Age, BMI, HRT, assessment centre, first five principal components	Main effects model	β coefficient=0.0022315	-0.0022983	0.0067614	3.34E-01

												Interaction model	β coefficient=0.0257468	-0.2146254	0.266119	8.30E-01
Metabolism Disorders	Letrlipid [taking letrozole and on lipid-altering medications]	*CYP19A1*	rs1008805	G	Additive	HDL cholesterol	Post-menopausal	Santa-Maria 2016	Age, BMI, race, change in plasma estradiol, and prior hormonal therapy and tamoxifen use	Mean Change, mg/dL (SE), P-value= Only Caucasian women: -5.01 (1.6), 0.003. Without adjusting for changes in plasma estradiol concentrations: -5.8 (1.6), 0.00054	Age, BMI, HRT, assessment centre, first five principal components	Main effects model	β coefficient=-0.0029017	-0.0058462	0.0000427	5.30E-02
												Interaction model	β coefficient=0.0410825	-0.1435086	0.2256737	6.60E-01
Metabolism Disorders	Letrlipid [taking letrozole and on lipid-altering medications]	*CYP19A1*	rs1008805	G	Dominant	HDL cholesterol	Post-menopausal	Santa-Maria 2016	Age, BMI, race, change in plasma estradiol, and prior hormonal therapy and tamoxifen use	Mean Change, mg/dL (SE), P-value= −6.6 (1.7), 0.00037. Only Caucasian women: -6.96 (2.2), 0.003	Age, BMI, HRT, assessment centre, first five principal components	Main effects model	β coefficient=-0.0048915	-0.0092489	-0.0005342	2.80E-02
												Interaction model	β coefficient=-0.0003716	-0.2550321	0.2542889	1.00E+00
Metabolism Disorders	Letrlipid [taking letrozole and on lipid-altering medications]	*CYP19A1*	rs749292	A	Dominant	HDL cholesterol	Post-menopausal	Santa-Maria 2016	Age, BMI, race, change in plasma estradiol, and prior hormonal therapy and tamoxifen use	Mean Change, mg/dL (SE), P-value= 9.5 (2.4), 0.0003. Only Caucasian women: 8.26 (2.1), 0.00035	Age, BMI, HRT, assessment centre, first five principal components	Main effects model	β coefficient=0.0017033	-0.0027562	0.0061628	4.50E-01
												Interaction model	β coefficient=0.0290638	-0.2113052	0.2694327	8.10E-01
Metabolism Disorders	Letrlipid [taking letrozole and on lipid-altering medications]	*CYP19A1*	rs10046	C	Additive	HDL cholesterol	Post-menopausal	Santa-Maria 2016	Age, BMI, race, change in plasma estradiol, and prior hormonal therapy and tamoxifen use	Mean Change, mg/dL (SE), P-value= −6.2 (1.6), 0.00046	Age, BMI, HRT, assessment centre, first five principal components	Main effects model	β coefficient=-0.0027602	-0.0056729	0.0001526	6.30E-02
												Interaction model	β coefficient=-0.0786367	-0.2607772	0.1035037	4.00E-01
Metabolism Disorders	Letrlipid [taking letrozole and on lipid-altering medications]	*CYP19A1*	rs2289105	T	Additive	HDL cholesterol	Post-menopausal	Santa-Maria 2016	Age, BMI, race, change in plasma estradiol, and prior hormonal therapy and tamoxifen use	Mean Change, mg/dL (SE), P-value= −6.2 (1.6), 0.00046	Age, BMI, HRT, assessment centre, first five principal components	Main effects model	β coefficient=-0.0028412	-0.0057554	0.000073	5.60E-02
												Interaction model	β coefficient=-0.0785562	-0.2606965	0.1035841	4.00E-01

Table S3 *Main-effect and treatment-genotype interaction analyses for binary MIADE outcomes in the UK Biobank*

System Organ Class	Regimen or Drug(s)	Gene	SNP rs# ID	Genotype	Statistical genetic model	Toxicity endpoint or Parameter	Menopausal status	Original study			Replication attempt in UK Biobank					
								Original study	Adjustement	Effect size [OR or HR (IC95%, UC95%), p-value]	Adjustment	Statistical model	OR	IC95%	UC95%	p_value
**Musculoskeletal Disorders**	**Anastrozole; Exemestane; Letrozole**	**CYP19A1**	**rs10046**	**A**	**Recessive**	**>5% bone loss (T-Score <= – o.5)**	**Post-menopausal**	**Baatjes 2020**	**N/R**	**OR=7.37 (1.101-49.336), p=0.04**	**Assessment centre, first five principal components**	**Main effects model**	**0.9493356**	**0.9253461**	**0.97395**	**6.80E-05**
												Interaction model	0.9675772	0.7322109	1.2786	8.20E-01
Musculoskeletal Disorders	Tamoxifen	CYP19A1	rs4646	A	Additive	Fractures & Osteoporosis	Post-menopausal	Leyland-Jones 2015 [1]	Age, BMI, smoking, HRT, bisphosphonates use, personal history of osteoporosis or bone fractures	HR= 0.80 (0.66, 0.99)	Age, BMI, smoking, HRT, Bisphosphonates, personal history of osteoporosis or bone fractures, assessment centre, first five principal components	Main effects model	1.04010448	0.9653562	1.12064	3.01E-01
												Interaction model	2.75883337	0.9777463	7.78439	5.52E-02
Musculoskeletal Disorders	Tamoxifen	CYP19A1	rs4646	A	Dominant	Fractures & Osteoporosis	Post-menopausal	Leyland-Jones 2015 [1]	Age, BMI, smoking, HRT, bisphosphonates use, personal history of osteoporosis or bone fractures	HR=0.76 (0.59, 0.98)	Age, BMI, smoking, HRT, Bisphosphonates, personal history of osteoporosis or bone fractures, assessment centre, first five principal components	Main effects model	1.00074542	0.911755	1.09842	9.87E-01
												Interaction model	3.46356567	0.6594666	18.1909	1.42E-01
Musculoskeletal Disorders	Tamoxifen	CYP19A1	rs10046	C	Dominant	Fractures & Osteoporosis	Post-menopausal	Leyland-Jones 2015 [1]	Age, BMI, smoking, HRT, bisphosphonates use, personal history of osteoporosis or bone fractures	HR=1.42 (1.05, 1.93)	Age, BMI, smoking, HRT, Bisphosphonates, personal history of osteoporosis or bone fractures, assessment centre, first five principal components	Main effects model	1.01784256	0.9180846	1.12844	7.37E-01
												Interaction model	1.20140861	0.2276916	6.3392	8.29E-01
Musculoskeletal Disorders	Tamoxifen	CYP19A1	rs10046	C	Additive	Fractures & Osteoporosis	Post-menopausal	Leyland-Jones 2015 [1]	Age, BMI, smoking, HRT, bisphosphonates use, personal history of osteoporosis or bone fractures	HR=1.28 (1.07, 1.52)	Age, BMI, smoking, HRT, Bisphosphonates, personal history of osteoporosis or bone fractures, assessment centre, first five principal components	Main effects model	1.0462356	0.9798895	1.11707	1.76E-01
												Interaction model	1.17220927	0.4223221	3.25362	7.60E-01
Musculoskeletal Disorders	Letrozole	CYP19A1	rs936308	G	Dominant	Fractures & Osteoporosis	Post-menopausal	Leyland-Jones 2015 [1]	Age, BMI, smoking, HRT, bisphosphonates use, personal history of osteoporosis or bone fractures	HR=0.73 (0.54, 0.99)	Age, BMI, smoking, HRT, Bisphosphonates, personal history of osteoporosis or bone fractures, assessment centre, first five principal components	Main effects model	1.06042402	0.9561191	1.17611	2.67E-01

												Interaction model	0.4908334	0.1064576	2.26304	3.60E-01
Musculoskeletal Disorders	Letrozole	ESR2	rs4986938	A	Dominant	Fractures & Osteoporosis	Post-menopausal	Leyland-Jones 2015 (2)	Age, BMI, smoking, HRT, bisphosphonates use, personal history of osteoporosis or bone fractures	HR=1.37 (1.01,1.84)	Age, BMI, smoking, HRT, Bisphosphonates, personal history of osteoporosis or bone fractures, assessment centre, first five principal components	Main effects model	0.97296801	0.8850735	1.06959	5.71E-01
												Interaction model	1.069043	0.3647232	3.13348	9.00E-01
Musculoskeletal Disorders	Letrozole	ESR1	rs2077647	C	Additive	Fractures & Osteoporosis	Post-menopausal	Leyland-Jones 2015 (2)	Age, BMI, smoking, HRT, bisphosphonates use, personal history of osteoporosis or bone fractures	HR=0.82 (0.68,0.99)	Age, BMI, smoking, HRT, Bisphosphonates, personal history of osteoporosis or bone fractures, assessment centre, first five principal components	Main effects model	0.97922223	0.9170176	1.04565	5.31E-01
												Interaction model	1.49561917	0.3785692	5.90876	5.66E-01
Musculoskeletal Disorders	Letrozole	ESR1	rs2077647	C	Dominant	Fractures & Osteoporosis	Post-menopausal	Leyland-Jones 2015 (2)	Age, BMI, smoking, HRT, bisphosphonates use, personal history of osteoporosis or bone fractures	HR=0.75 (0.58,0.98)	Age, BMI, smoking, HRT, Bisphosphonates, personal history of osteoporosis or bone fractures, assessment centre, first five principal components	Main effects model	1.00131423	0.9020355	1.11152	9.80E-01
												Interaction model	1.10078559	0.1099067	11.0251	9.35E-01
Musculoskeletal Disorders	Anastrozole; Letrozole	CYP19A1	rs4646	G	Recessive	Osteoporosis	Post-menopausal	Mazzuca 2016	Age	OR=36.63 (1.99-673.8), P = 0.0154 [Total sample n=11 (24.4)/(n=45), GG n=11 (45.8)/(n=24), GT n=0 (0.0)/(n=19), TT n=0 (0.0)/(n=2), 0.001]	Age, assessment centre, first five principal components [those who are positive for osteoporotic, before the therapy, being excluded]	Main effects model	0.98468453	0.9093068	1.06631	7.04E-01
												Interaction model	0.77122364	0.3388614	1.75525	5.36E-01
Musculoskeletal Disorders	Anastrozole; Letrozole	TNFRSF11B	rs2073618	C	Recessive	Osteoporosis [<−2.5]	Post-menopausal	Wang 2015	N/R	OR=2.76 (1.13, 6.73), P = 0.0259 [CC n (%) vs. GG n (%), p-value: 18 (36.0)/(n=50) vs. 10(16.9)/(n=59), 0.029]	Assessment centre, first five principal components	Main effects model	1.08332729	0.983686	1.19306	1.04E-01
												Interaction model	0.62974768	0.2417269	1.64062	3.44E-01
Musculoskeletal Disorders	Anastrozole; Letrozole	TNFSF11	rs7984870	C	Recessive	Osteoporosis [<−2.5]	Post-menopausal	Wang 2015	N/R	OR=4.19 (1.16, 15.17), P = 0.0288 [CC n (%) vs GG n (%), p-value = 23 (30.2)/(n=76) vs. 3 (9.4)/(n=32), 0.026]	Assessment centre, first five principal components	Main effects model	0.96696838	0.876096	1.06727	5.05E-01
												Interaction model	0.77266041	0.3176162	1.87964	5.70E-01

**Musculoskeletal Disorders**	**Anastrozole; Letrozole**	**TNFRSF11B**	**rs2073618**	**C**	**Recessive**	**Osteopenia [−2.5 –−1.0]**	**Post-menopausal**	**Wang 2015**	**N/R**	**OR=2.61 (1.12-6.11), P = 0.0268 [CC n (%) vs. GG n (%), p-value = 20 (40.0)/(n=50) vs. 12 (20.3)/(n=59), 0.034]**	**Assessment centre, first five principal components**	**Main effects model**	**1.073256**	**1.043746**	**1.1036**	**6.70E-07**
												Interaction model	1.17818	0.856125	1.62139	3.10E-01
Musculoskeletal Disorders	Anastrozole; Letrozole	TNFSF11	rs7984870	C	Recessive	Osteopenia [−2.5 –−1.0]	Post-menopausal	Wang 2015	N/R	OR=3.15 (1.16, 8.55), P = 0.0241 [CC n (%) vs. GG n (%), p-value = 32 (42.1)/(n=76) vs. 6 (18.8)/(n=32), 0.027]	Assessment centre, first five principal components	Main effects model	1.013846	0.9860768	1.0424	3.30E-01
												Interaction model	0.9206356	0.6726205	1.2601	6.10E-01
**Musculoskeletal Disorders**	**Anastrozole; Letrozole**	**TNFRSF11B**	**rs2073618**	**C**	**Recessive**	**T-score [>-1.0 SD (Normal)]**	**Post-menopausal**	**Wang 2015**	**N/R**	**OR=0.1878 (0.0813, 0.4334), P = 0.0001 [CC n (%) vs. GG n (%), p-value = 12 (24.0)/(n=50) vs. 37 (62.7)/(n=59), 9.20E-5]NOT 0.38 (0.23; 0.65)**	**Assessment centre, first five principal components**	**Main effects model**	**0.9270607**	**0.9018328**	**0.95299**	**7.40E-08**
												Interaction model	0.9004484	0.6552269	1.23745	5.20E-01
Musculoskeletal Disorders	Anastrozole; Letrozole	TNFSF11	rs7984870	C	Recessive	T-score [>-1.0 SD (Normal)]	Post-menopausal	Wang 2015	N/R	OR=0.1494 (0.0595 to 0.3750), P = 0.0001 [CC n (%) vs. GG n (%), p-value = 21 (27.2)/(n=76) vs. 23 (71.8)/(n=32), 3.15E -5] NOT 0.38 (0.25, 0.59)	Assessment centre, first five principal components	Main effects model	0.9891221	0.96231	1.01668	4.40E-01
												Interaction model	1.123564	0.8234567	1.53305	4.60E-01
Musculoskeletal Disorders	Anastrozole; Letrozole	TNFSF11	rs7984870	C	Additive	Musculoskeletal Adverse Events	Post-menopausal	Wang 2015	N/R	OR=1.787 (1.359–2.351), p=3.07E-5	Assessment centre, first five principal components	Main effects model	1.01350595	0.7835577	1.31094	9.19E-01
												Interaction model	1.25497007	0.0738986	21.3123	8.75E-01
Musculoskeletal Disorders	Anastrozole; Letrozole	TNFSF11	rs7984870	C	Recessive	Musculoskeletal Adverse Events	Post-menopausal	Wang 2015	N/R	OR=3.259 (1.843–5.763), p=2.19E-4	Assessment centre, first five principal components	Main effects model	0.84278578	0.5254949	1.35166	4.78E-01
												Interaction model	1 (omitted)			
Musculoskeletal Disorders	Anastrozole; Letrozole	TNFRSF11B	rs2073618	C	Additive	Musculoskeletal Adverse Events	Post-menopausal	Wang 2015	N/R	OR=1.623 (1.233–2.141), p=6.09E-4	Assessment centre, first five principal components	Main effects model	0.9461669	0.7303715	1.22572	6.75E-01
												Interaction model	1.42743124	0.079961	25.4819	8.09E-01

Musculoskeletal Disorders	Anastrozole; Letrozole	TNFRSF11B	rs2073618	C	Recessive	Musculoskeletal Adverse Events	Post-menopausal	Wang 2015	N/R	OR=2.931 (1.624–5.288), p=7.95E-4	Assessment centre, first five principal components	Main effects model	0.72068008	0.43605	1.1911	2.01E-01
												Interaction model	1 (omitted)			
Musculoskeletal Disorders	Anastrozole; Letrozole	ESR1	PvuII (rs2234693)	T	Additive	Musculoskeletal Adverse Events	Post-menopausal	Wang 2013	Age, type of third generation AIs, BMI and prior tamoxifen use	OR=0.510 (0.389–0.669), p=9.49E-07	Age, BMI, assessment centre, first five principal components	Main effects model	0.93662941	0.7243718	1.21108	6.18E-01
												Interaction model	convergence not achieved			
Musculoskeletal Disorders	Anastrozole; Letrozole	ESR1	PvuII (rs2234693)	T	Recessive	Musculoskeletal Adverse Events	Post-menopausal	Wang 2013	Age, type of third generation AIs, BMI and prior tamoxifen use	OR=0.282 (0.147–0.540), p=0.001	Age, BMI, assessment centre, first five principal components	Main effects model	0.90766325	0.6043561	1.36319	6.41E-01
												Interaction model	convergence not achieved			
Musculoskeletal Disorders	Anastrozole; Letrozole	ESR1	XbaI (rs9340799)	G	Additive	Musculoskeletal Adverse Events	Post-menopausal	Wang 2013	Age, type of third generation AIs, BMI and prior tamoxifen use	OR=0.553 (0.399–0.764), p=3.09E-04	Age, BMI, assessment centre, first five principal components	Main effects model	0.92033887	0.7012185	1.20793	5.50E-01
												Interaction model	1 (omitted)			
Musculoskeletal Disorders	Anastrozole; Letrozole	ESR1	XbaI (rs9340799)	G	Recessive	Musculoskeletal Adverse Events	Post-menopausal	Wang 2013	Age, type of third generation AIs, BMI and prior tamoxifen use	OR=0.593 (0.441–0.797)	Age, BMI, assessment centre, first five principal components	Main effects model	0.96064996	0.5495118	1.6794	8.88E-01
												Interaction model	1 (omitted)			
Hepatobiliary Disorders	Tamoxifen	CYP17A1	CYP17 A1 allele (rs743572)	A	Additive	Hepatic steatosis	Pre- , Peri- & Post-menopausal	Ohnishi 2005	N/R	OR=0.53 (0.33-0.82), p=0.005	Assessment centre, first five principal components	Main effects model	0.99484593	0.9446191	1.04774	8.45E-01
												Interaction model	1.1687376	0.6481178	2.10756	6.04E-01
Hepatobiliary Disorders	Tamoxifen	CYP17A1	CYP17 A2 allele (rs743572)	G	Additive	Hepatic steatosis	Pre- , Peri- & Post-menopausal	Ohnishi 2005	N/R	OR=1.90 (1.21-2.99)	Assessment centre, first five principal components	Main effects model	1.00518077	0.9544321	1.05863	8.45E-01
												Interaction model	0.85562405	0.4744822	1.54293	6.04E-01
Hepatobiliary Disorders	Tamoxifen	CYP17A1	CYP17 A2/A2 genotype (rs743572)	G	Recessive	Hepatic steatosis	Pre- , Peri- & Post-menopausal	Ohnishi 2005	N/R	OR=3.60 (1.42–9.10)	Assessment centre, first five principal components	Main effects model	0.99479172	0.8985591	1.10133	9.20E-01
												Interaction model	0.24444076	0.0327627	1.82376	1.69E-01
Hepatobiliary Disorders	Tamoxifen	CYP2D6	CYP2D6*41 [combination of 2988G>A (rs28371725), 2850C>T (rs16947), –1584C (rs1080985)]	A, T, C	Additive	Hepatic steatosis	Pre- , Peri- & Post-menopausal	Wickramage 2017	N/R	(P=0.029)	Assessment centre, first five principal components	Main effects model	1 (omitted)			
												Interaction model	1 (omitted)			

**Vascular Disorders**	**Tamoxifen**	**F5**	**FV Leiden (rs6025)**	**A**	**Dominant**	**Thromboembolic events**	**Pre- , Peri- & Post-menopausal**	**Garber 2010**	**Smoking, personal history and family history of thromboembolic events**	**OR=4.73 (2.10, 10.68), p < .001**	**Assessment centre, first five principal components, smoking, family history of stroke**	**Main effects model**	**1.617275**	**1.426763**	**1.83323**	**5.60E-14**
												Interaction model	1.954492	0.7824797	4.88197	1.50E-01
**Vascular Disorders**	**Tamoxifen**	**F5**	**FV Leiden (rs6025)**	**A**	**Dominant**	**Venous thromboembolism [DVT/PE]**	**Pre- , Peri- & Post-menopausal**	**Kovac 2015**	**N/R**	**OR=3.32 (1.18, 9.35), P = 0.023 [VTE and FV Leiden mutation frequency (10/50 vs 7/100; p=0.020)]**	**Assessment centre, first five principal components**	**Main effects model**	**1.403962**	**1.184609**	**1.66393**	**9.10E-05**
												Interaction model	3.017497	1.092581	8.33375	3.30E-02
**Vascular Disorders**	**Tamoxifen**	**F5, F2**	**FV Leiden (rs6025) or FII G 20210A (rs1799963)**	**A, A**	**Dominant**	**Venous thromboembolism [DVT/PE]**	**Pre- , Peri- & Post-menopausal**	**Kovac 2015**	**N/R**	**OR=3.50 (1.42, 8.60), P = 0.0063 [Prothrombotic mutations and VTE (14/50 vs 10/100; p=0.009)]**	**Assessment centre, first five principal components**	**Main effects model**	**1.54**	**1.35**	**1.77**	**4.10E-10**
												Interaction model	2.88	1.18	7.03	2.0e-02
Vascular Disorders	Tamoxifen	ESR1	XbaI (rs9340799)	G	Dominant	Venous thromboembolism [DVT/PE]	Pre- , Peri- & Post-menopausal	Onitilo 2009	Unadjusted analysis but the association persisted after adjusting for classical risk factors including age at diagnosis and body mass index at enrollment	HR=3.47 (0.97–12.44), p=0.035	Assessment centre, first five principal components	Main effects model	1.00775721	0.9283208	1.09399	8.54E-01
												Interaction model	1.10882431	0.1372882	8.95555	9.23E-01
Reproductive System	Tamoxifen	CYP3A4	CYP3A4*1B (rs2740574)	C	Additive	Endometrial cancer	Pre- , Peri- & Post-menopausal	Chu 2007	N/R	OR=2.8 (1.4, 5.8), p=0.004	Assessment centre, first five principal components	Main effects model	0.87133401	0.6779862	1.11982	2.82E-01
												Interaction model	1 (omitted)			
Reproductive System	Tamoxifen	ABCB1	rs1045642	T	Additive	Gynaecological AEs [endometrial hyperplasia or cancer]	Pre- , Peri- & Post-menopausal	Argalacsova 2017	N/R	HR=1.0588, p=0.0221: v/v, n/total=19/51, wt/v, n/total=21/127, Wt/wt, n/total=13/72	Assessment centre, first five principal components	Main effects model	1.05371379	0.9816733	1.13104	1.48E-01
												Interaction model	0.91455654	0.5804361	1.44101	7.00E-01

Reproductive System	Tamoxifen	CYP3A5	CYP3A5*3 (rs776746)	G	Dominant	Endometrial hyperplasia	Pre- , Peri- & Post-menopausal	Miranda 2021	BMI, blood group, smoking habit, socioeconomic status, treatment with oral contraceptives, hormone replacement therapies, age at menarche, menopausal status, family history of cancer, age at diagnosis, cancer stage	OR=0.067 (0.009–0.524), p=0.010	Age, BMI, smoking, HRT, menopausal status, age at menarche, family history of cancer, oral contraceptives, assessment centre, first five principal components	Main effects model	0.66590009	0.2130451	2.08136	4.84E-01
												Interaction model	101334.3	0	.	9.90E-01
Reproductive System	Tamoxifen	CYP3A5	CYP3A5*3 (rs776746)	G	Recessive	Endometrial hyperplasia	Pre- , Peri- & Post-menopausal	Miranda 2021	BMI, blood group, smoking habit, socioeconomic status, treatment with oral contraceptives, hormone replacement therapies, age at menarche, menopausal status, family history of cancer, age at diagnosis, cancer stage	OR=0.208 (0.047–0.758), p=0.007	Age, BMI, smoking, HRT, menopausal status, age at menarche, family history of cancer, oral contraceptives, assessment centre, first five principal components	Main effects model	0.93744399	0.7148078	1.22942	6.41E-01
												Interaction model	1656736	0	.	9.90E-01
Reproductive System	Tamoxifen	CYP2D6	CYP2D6*4 (rs1800716)	A	Additive	Endometrial hyperplasia	Post-menopausal	Dieudonné 2014	N/R	HR=1.88 (1.28–2.77), p=0.0022	Assessment centre, first five principal components	Main effects model	1.17170031	0.9540495	1.439	1.31E-01
												Interaction model	1.15565318	0.3068843	4.35191	8.31E-01
Reproductive System	Tamoxifen	CYP2D6	CYP2D6*4 (rs1800716)	A	Recessive	Endometrial hyperplasia	Post-menopausal	Dieudonné 2014	N/R	HR=2.786 (0.99–7.86), p=0.0022	Assessment centre, first five principal components	Main effects model	0.96108166	0.5247483	1.76023	8.98E-01
												Interaction model	5.53506273	0.57215	53.547	1.39E-01
Reproductive System	Anastrozole; Exemestane; Letrozole	ESR1	Xbal (rs9340799)	A	Recessive	Endometrial hyperplasia	Post-menopausal	Koukouras 2012	N/R	Mean ± SD: Xbal (AA)= –4.27 ± 3.3 vs. Xbal (AG+GG)=−1.73 ± 2.2 [p=0.005]	Assessment centre, first five principal components	Main effects model	0.81085152	0.6267122	1.04909	1.11E-01
												Interaction model	1 (omitted)			
Psychiatric Disorders	Tamoxifen	UGT2B7	UGT2B7*2 (rs7439366)	T	Additive	Depression	Pre- , Peri- & Post-menopausal	Al-Mamun 2017	Age, BMI	OR=2.61, p=0.033	Age, BMI, assessment centre, first five principal components	Main effects model	0.99783881	0.9662089	1.0305	8.95E-01
												Interaction model	0.92603434	0.6140076	1.39663	7.14E-01
Psychiatric Disorders	Tamoxifen	UGT2B7	UGT2B7*2 (rs7439366)	T	Additive	Depression	Pre- , Peri- & Post-menopausal	Al-Mamun 2017	Unadjusted	OR=2.5666 (1.0740- 6.1337)	Assessment centre, first five principal components	Main effects model	0.99997132	0.9684329	1.03254	9.99E-01
												Interaction model	0.90597373	0.6003133	1.36727	6.38E-01
Psychiatric Disorders	Tamoxifen	CYP2D6	CYP2D6*4 (rs1800716)	A	Additive	Depression	Pre- , Peri- & Post-menopausal	Al-Mamun 2017	Age, BMI	OR=0.078, p=0.001	Age, BMI, assessment centre, first five principal components	Main effects model	1.01626315	0.9773365	1.05674	4.18E-01

												Interaction model	1.1550808	0.7115666	1.87503	5.60E-01
Psychiatric Disorders	Tamoxifen	CYP2D6	CYP2D6*4 (rs1800716)	A	Additive	Depression	Pre- , Peri- & Post-menopausal	Al-Mamun 2017	Unadjusted	OR=0.0835 (0.0197- 0.3551)	Assessment centre, first five principal components	Main effects model	1.01367287	0.9750603	1.05381	4.93E-01
												Interaction model	1.17033815	0.7196254	1.90334	5.26E-01
Psychiatric Disorders	Tamoxifen	CYP2D6	CYP2D6*4 (rs1800716)	A	Dominant	Depression	Pre- , Peri- & Post-menopausal	Al-Mamun 2017	Age, BMI	OR=0.074, p=0.0006	Age, BMI, assessment centre, first five principal components	Main effects model	1.02196733	0.9750678	1.07112	3.65E-01
												Interaction model	1.27080541	0.7010576	2.30359	4.30E-01
Psychiatric Disorders	Tamoxifen	CYP2D6	CYP2D6*4 (rs1800716)	A	Dominant	Depression	Pre- , Peri- & Post-menopausal	Al-Mamun 2017	Unadjusted	OR=0.0789 (0.0186, 0.3353)	Assessment centre, first five principal components	Main effects model	1.01862956	0.9721575	1.06732	4.38E-01
												Interaction model	1.27482839	0.7040252	2.30842	4.23E-01
Psychiatric Disorders	Tamoxifen	CYP2D6	CYP2D6*10 (rs1065852)	T	Additive	Depression	Pre- , Peri- & Post-menopausal	Al-Mamun 2017	Age, BMI	OR=1.77E+08, p=0.0223	Age, BMI, assessment centre, first five principal components	Main effects model	1.01567489	0.9782344	1.05455	4.17E-01
												Interaction model	1.247782	0.7887496	1.97396	3.40E-01
Psychiatric Disorders	Tamoxifen	CYP2D6	CYP2D6*10 (rs1065852)	T	Additive	Depression	Pre- , Peri- & Post-menopausal	Al-Mamun 2017	Unadjusted	OR=27.9818 (1.6064, 487.4241)	Assessment centre, first five principal components	Main effects model	1.01272692	0.9755943	1.05127	5.07E-01
												Interaction model	1.2701942	0.8022887	2.01099	3.08E-01
Psychiatric Disorders	Tamoxifen	CYP2D6	CYP2D6*10 (rs1065852)	T	Recessive	Depression	Pre- , Peri- & Post-menopausal	Al-Mamun 2017	Age, BMI	OR=2.32E+08, p=0.0117	Age, BMI, assessment centre, first five principal components	Main effects model	1.00886723	0.9156496	1.11157	8.58E-01
												Interaction model	1.081103	0.3235373	3.61252	9.00E-01
Psychiatric Disorders	Tamoxifen	CYP2D6	CYP2D6*10 (rs1065852)	T	Recessive	Depression	Pre- , Peri- & Post-menopausal	Al-Mamun 2017	Unadjusted	OR=37.2020 (2.2369- 618.7153)	Assessment centre, first five principal components	Main effects model	1.00504463	0.9126214	1.10683	9.19E-01
												Interaction model	1.14336845	0.3433103	3.8079	8.27E-01
Psychiatric Disorders	Tamoxifen	CYP2D6	CYP2D6*10 (rs1065852)	T	Dominant	Depression	Pre- , Peri- & Post-menopausal	Al-Mamun 2017	Age, BMI	OR=2.15E+08, p=0.0140	Age, BMI, assessment centre, first five principal components	Main effects model	1.02205069	0.9755618	1.07075	3.58E-01
												Interaction model	1.397593	0.7752137	2.51965	2.70E-01
Psychiatric Disorders	Tamoxifen	CYP2D6	CYP2D6*10 (rs1065852)	T	Dominant	Depression	Pre- , Peri- & Post-menopausal	Al-Mamun 2017	Unadjusted	OR=33.5350 (2.0359, 552.3914)	Assessment centre, first five principal components	Main effects model	1.01838464	0.9723326	1.06662	4.40E-01
												Interaction model	1.41390376	0.7850749	2.54641	2.49E-01

Table S4 *Post hoc power to detect genotype-endocrine therapy interaction term (per additional minor allele) in a logistic regression model; $\alpha=0.05$ and $\alpha=5.15\times10^{-4}$)*

a. For rare variants and rare events

Gene	SNV ID	Variant (MAF)	Endpoint (events in endocrine users)	Menopausal status	Expected OR	Assumed interaction OR*	Estimated power range (α=0.05 (conventional, two-sided)	Estimated power range ($\alpha=5.15\times10^{-4}$ Bonferroni)
F2	rs1799963 *(F2 FII G20210A)*	0.01	Thromboembolic events (n=99)	Pre- , Peri- & Post-menopausal	OR= ~3 -4	3×	63.7%	12.3%
						5×	**97.9%**	70%
						10×	**~100%**	**99.9%**
F5	rs6025 *(FVL)*	0.02	Thromboembolic events (n=99)	Pre- , Peri- & Post-menopausal	OR= ~3 -4	3×	**88.9%**	38.5%
						5×	**99.9%**	**97.6%**
						10×	**~100%**	**~100%**
CYP3A4	rs2740574	0.03	Endometrial cancer (n=35)	Pre- , Peri- & Post-menopausal	OR= ~2 -3	3×	65%	13%
						5×	**98%**	73%
						10×	**100%**	**100%**

**OR denotes the odds ratio for the genotype×endocrine therapy interaction term under an additive genotype model (per additional minor allele).*

b. For representative common variants and higher-frequency outcomes

Gene	SNV ID	Variant (MAF)	Endpoint (events in endocrine users)	Menopausal status	Expected OR	Assumed interaction OR*	Estimated power range (α=0.05 (conventional, two-sided)	Estimated power range ($\alpha=5.15\times10^{-4}$ Bonferroni)
UGT2B7	rs7439366	0.46	Depression (n=117)	Pre- , Peri- & Post-menopausal	OR= ~2 -3	1.5	**94.64%**	53.90%
						2	**100%**	**99.96%**
						3×	**100%**	**100%**
						5×	**100%**	**100%**
						10×	**100%**	**100%**
ESR1	rs9340799	0.35	Venous thromboembolism (DVT/PE) (n=66)	Pre- , Peri- & Post-menopausal	OR= ~3 -4	1.5	73.75%	19.02%
						2	**99.88%**	**93.59%**
						3×	**100%**	**100%**
						5×	**100%**	**100%**
						10×	**100%**	**100%**

**OR denotes the odds ratio for the genotype×endocrine therapy interaction term under an additive genotype model (per additional minor allele).*

Table S5 *Endocrine agents and other medications codes used in the UK Biobank analysis.*

Treatment modality	Generic name	Brand names	Codes*
Endocrine Therapy	Tamoxifen	TAMOXIFEN, EMBLON, NOLTAM, NOLVADEX, Soltamox, tamofen	1140870164, 1140870182, 1140858348, 1140870170, 1140870176, 1141170264
	Letrozole	LETROZOLE, FEMARA	1141145896, 1141145900
	Anastrozole	ANASTROZOLE, ARIMIDEX	1140923018, 1140923022
	Exemestane	EXEMESTANE, AROMASIN	1141171100, 1141171104
Bone Antiresorptive Therapy	Alendronate sodium	Fosamax, Binosto, Fosavance, Bentexo	1140922174, 1141176570
	Ibandronic acid	Bondronat, Iasibon, Bonviva, Quodixor	1141180314, 1141190534
	Pamidronate disodium		1140868784
	Risedronate sodium	Actonel	1141175684, 1141175690
	Zoledronic acid	Zometa, Aclasta, Zerlinda	1141173814
	Teriparatide	Forsteo, Movymia, Terrosa	1141188794, 1141188798
	Raloxifene	Evista	1141168574, 1141168578
	Denosumab	Prolia, Xgeva	N/A
	Romosozumab	Evenity	N/A
Lipid-lowering Therapy	Lipid-lowering drug (generic)		1140861922
	Simvastatin	Zocor, zocor heart-pro, INEGY	1140861958, 1140881748, 1141200040
	Pravastatin	Lipostat	1140888648, 1140861970
	Rosuvastatin	Crestor	1141192410, 1141192414
	Fluvastatin	Lescol	1140888594, 1140864592
	Atorvastatin	Lipitor	1141146234, 1141146138
	Ezetimibe	Ezetrol	1141192736, 1141192740

	Acipimox	Olbetam	1140861892, 1140861894
	Bezafibrate/Bezafibrate product	Bezalip, Fibrazate	1140861924, 1141157260, 1141201306, 1140861926, 1140861928
	Ciprofibrate		1140862026
	Fenofibrate	Supralip, Lipantil	1140861954, 1141172214, 1141162544
	Gemfibrozil/Gemfibrozil product	Lopid	1140861856, 1141157262, 1140861858
	Colestipol	Colestid	1140888590, 1140861848
	Cholestyramine/Cholestyramine product	Questran	1140909780, 1141180722, 1141180734, 1140861936
	Clofibrate		1140861944

*Treatments and medication codes (including those used as used as covariates) were obtained from:
https://biobank.ctsu.ox.ac.uk/crystal/coding.cgi?id=4&nl=1
https://biobank.ndph.ox.ac.uk/showcase/field.cgi?id=20003

Table S6 *Ascertainment of biomarkers and other phenotypes at the baseline*

A. Cholesterol, LDL, HDL and Triglycerides biomarkers at the baseline

Description	Data-Field	Items of Data/Participants	Units of measurement	Measurement method
Cholesterol	30690	487,377/470,756	mmol/L	Measured by CHO-POD analysis on a Beckman Coulter AU5800
LDL direct	30780	486,454/469,918	mmol/L	Measured by enzymatic protective selection analysis on a Beckman Coulter AU5800
HDL cholesterol	30760	429,793/429,793	mmol/L	Measured by enzyme immunoinhibition analysis on a Beckman Coulter AU5800
Triglycerides	30870	486,969/470,386	mmol/L	Measured by GPO-POD analysis on a Beckman Coulter AU5800

B. Bone Mineral Denisty, T-score, Osteoporosis and Osteopenia Variables at the baseline

1) Bone Mineral Denisty
Bone Mineral Density (BMD) g/cm2. The Heel bone mineral density (BMD):
https://biobank.ndph.ox.ac.uk/ukb/field.cgi?id=3148
Method: Heel ultrasound method (left). Bone-densitometry of heel:
https://biobank.ndph.ox.ac.uk/ukb/field.cgi?id=4092
This uses BMD and if it is missing, we used Heel bone mineral density (BMD), manual entry:
https://biobank.ndph.ox.ac.uk/ukb/field.cgi?id=3084
And if this was missing, we used Heel bone mineral density (BMD) (left) or Heel bone mineral density (BMD) (right):
https://biobank.ndph.ox.ac.uk/ukb/field.cgi?id=4105
https://biobank.ndph.ox.ac.uk/ukb/field.cgi?id=4124

The following data Field IDs were used for **BMD**:
3148 Heel bone mineral density (BMD)
4105 Heel bone mineral density (BMD) (left)
4124 Heel bone mineral density (BMD) (right)
3084 Heel bone mineral density (BMD), manual entry

2) T-score
T-score (number of SD BMD above or below standard) was computed from **Heel bone mineral density (BMD) T-score, automated** (bone-densitometry of heel). The following data Field IDs were used:
78 Heel bone mineral density (BMD) T-score, automated
4125 Heel bone mineral density (BMD) T-score, automated (right)
4106 Heel bone mineral density (BMD) T-score, automated (left)
4138 Heel bone mineral density (BMD) T-score, manual entry (left)
4143 Heel bone mineral density (BMD) T-score, manual entry (right)

3) Osteoporosis and Osteopenia
Osteoporosis and Osteopenia were calculated based on **Heel bone mineral density (BMD) T-score** as follows:
Osteopenia: T score less than or equal to -1 but greater than -2.5
Osteoporosis: T score less than or equal to -2.5

Table S7 *Ascertainment of incident phenotypic endpoints of adverse drug effects and other variables used in the UK Biobank analysis.*

A. Incident phenotypic endpoints of adverse drug effects and other phenotypes used as co-variates

Phenotype	ICD10	ICD9	Self-report code from n_20002_* variable

**Scoliosis (including Kyphoscoliosis)**	M41 M965 Q763 Q675	7373 75420	1535
**Osteoporosis**	M80 M81 M82	7330	1309
**Fractures**	M484 M840 M841 M842 M843 M844 S02 S12 S220 S222 S223 S224 S228 S229 S32 S42 S52 S62 S72 S82 S92 T02 T08 T10 T12 T14 M800 M801 M804 M805 M808 M809	80 81 82 7331 E8879	1626 1627 1628 1629 1630 1631 1632 1633 1634 1635 1636 1637 1638 1639 1640 1644 1645 1646 1647 1648 1649 1650 1651 1652 1653 1654 1655 1656
**Severe musculoskeletal adverse events (e.g., myopathy, myositis)**	G720 G728 G729 M608 M609 M814	3599	1322
**Thromboembolic events**	I82 I26 I801 I802 I803 I808 I809 I81 I82 I630 I631 I633 I634 I74 K550 I82 I513 I676 I636 I240	453 4151 4341 444 59381 3259 4340 4376 4511 4512 4518 4519 4529 59382	1094 1093 1088 1068
**Venous thromboembolism (DVT/PE)**	I82 I26 I513 I676 I636 I240	453 4151 3259 4340 4376 4511 4512 4518 4519 4529 59382	1094 1093 1068
**Hepatosteatosis**	K76 K760 K758	5715 5716 5718 5719	
**Gynaecological events [endometrial thickening, uterine fibroids, ovarian cysts, adenomyosis and cervical canal cysts]**	N850 N851 D25 N830 N832 N800 Q516	6213 2191 2360 218 6200 6201 6202	1351 1349
**Gynaecological events [endometrial hyperplasia or endometrial cancer]**	N850 N851 C541	6213 2191 2360 182	1040
**Endometrial hyperplasia or Double endometrial thickness**	N850 N851	6213 2191 2360	
**Depression**	F33 F32 F412 F341	3004 311	1286
**Varicose veins**	I83 I86	454 456	1494
**Endometrial cancer**	C541	182	1040
**Prosthetic limb by prosthesis**	Z441 Z971	V437 V520	
**Chemotherapy**	Z082 Z511 Z542 Z926	V581 V662 V6621 V6622 V6629 V672	
**Radiotherapy**	Z081 Z091 Z510	V580 V661 V6611 V6612 V6619 V671	
**Family history of ischaemic heart disease**	Z824	V173	
**Family history of stroke**	Z823	V171	
**Family history of musculoskeletal diseases or arthritis**	Z826	V177 V178	
**Family history of malignant neoplasm of genital organs**	Z804	V164	
**Family history of genital disease**	Z804 Z842	V187 V164	
**Family history of psychiatric and other mental and behavioural disorders**	Z818	V170	
**Family history of blood disease**	Z832	V183	

B. Family history variables of cancer, depression or stroke used as co-variates

Data field IDs and codes for relevant illnesses of father/mother/siblings were used

Field ID for illnesses of father/mother/siblings:
20107 Illnesses of father
20110 Illnesses of mother
20111 Illnesses of siblings

Coding for illnesses of father/mother/siblings:
2 Stroke
3 Lung cancer
4 Bowel cancer
5 Breast cancer
13 Prostate cancer
12 Severe depression

C. Menopausal status

Menopausal status was determined at the baseline UK Biobank visit:
0=premenopausal
1=postmenopausal due to natural menopause
2=postmenopausal due to surgery i.e., hysterectomy or oophorectomy
3=postmenopausal but can't tell what type of menopause or age at menopause as taking HRT over age at menopause
4=premenopausal but taking HRT so can't be sure
9=missing data for one or more variables so can't define this.

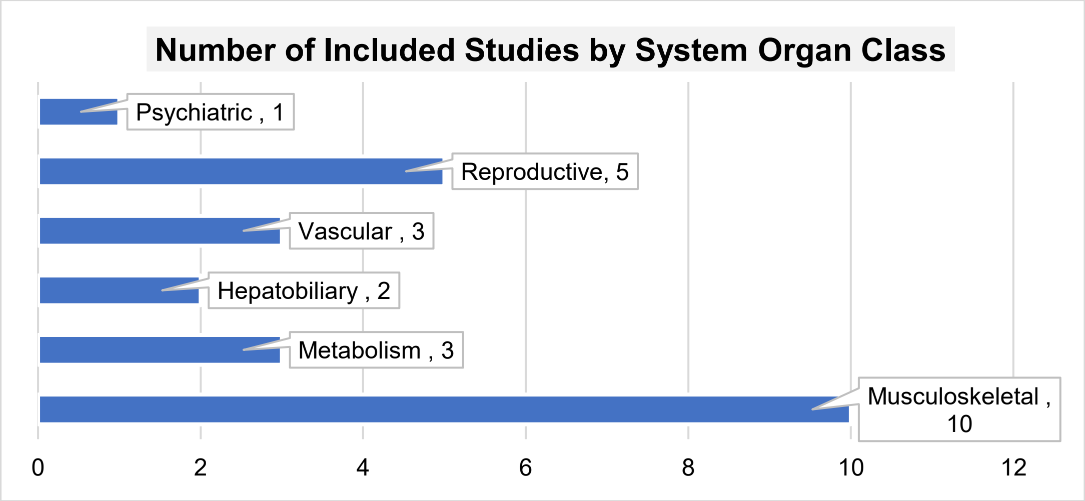

Figure S1 *Number of identified PGx studies of ADEs related to endocrine therapy by system organ class.*

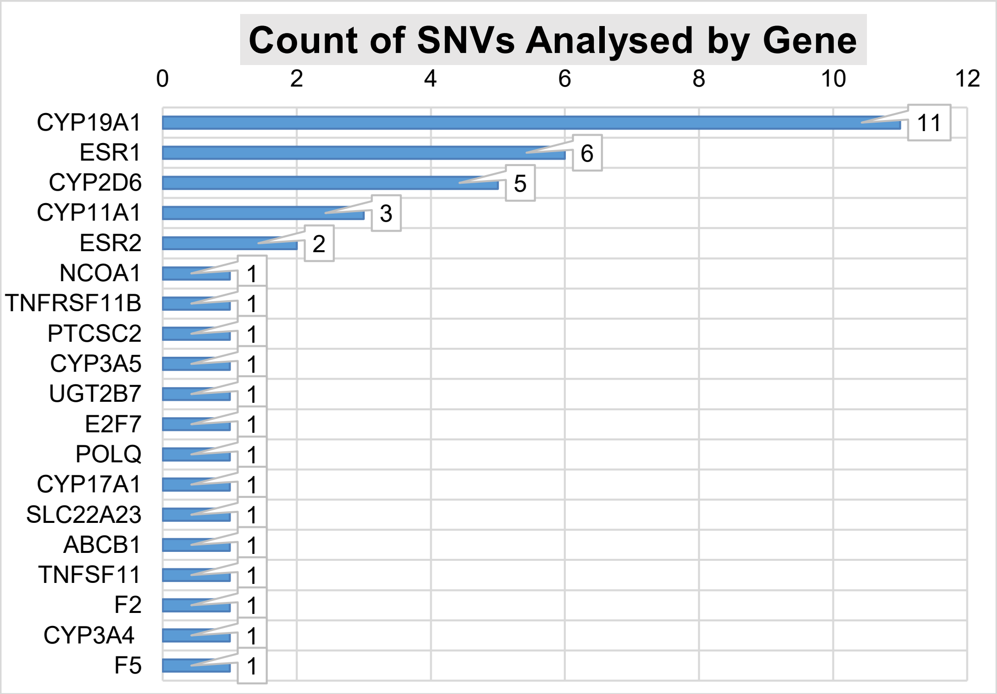

Figure S2 *Count of SNVs analysed in the UK Biobank per gene regarding PGx studies of ADEs related to endocrine therapy.*
